# Functional and structural basis of a hypermorphic TRPC3 variant

**DOI:** 10.1126/sciadv.aec9284

**Published:** 2026-03-25

**Authors:** Briar Bell, Angela M. Jaramillo-Granada, Luis O. Romero, Irene A. Gutierrez, Venkata K.P.S. Mallampalli, Guizhen Fan, Sameer Varma, Matthew L. Baker, Irina I. Serysheva, Valeria Vásquez, Julio F. Cordero-Morales

**Affiliations:** ^1^ Department of Biochemistry and Molecular Biology, Center for Membrane Biology, McGovern Medical School at the University of Texas Health Science Center at Houston, Houston, TX, USA.; ^2^ Department of Molecular Biosciences, University of South Florida, Tampa, FL, USA.; ^3^ Structural Biology Imaging Center at the University of Texas Health Science Center at Houston, Houston, TX, USA.

## Abstract

Cerebellar ataxias are characterized by impaired motor coordination resulting from neuronal dysfunction within the cerebellum. The mechanisms underlying this pathology and its cerebellar-specific neurodegeneration remain unknown. We uncover how a gain-of-function canonical transient receptor potential member 3 (TRPC3) mutation, coupled with a cerebellum-specific isoform, stabilizes the channel’s open state, resists the leading inhibitor Pyr3, and drives calcium-dependent cell death. Restoring calcium homeostasis by expressing a Purkinje cell calcium pump improves cell viability. Transgenic expression of the TRPC3 hypermorphic variant in *Caenorhabditis elegans* induces neurodegeneration, confirming its pathogenicity across species. Cryo–electron microscopy and molecular simulations reveal the structural basis for the stabilization of the cerebellar-specific TRPC3 variant in its open state and uncover a druggable allosteric inhibitory binding site. These findings provide an explanation for the vulnerability of cerebellar neurons in TRPC3-associated ataxias and highlight a site for therapeutic intervention.

## Teaser

Structural snapshots capture an ataxia-linked overactive ion channel in its open state and reveal a druggable site for inhibition.

## INTRODUCTION

Cerebellar ataxia is a condition characterized by impaired motor coordination resulting from degeneration of the cerebellum, leading to poor balance and gait abnormalities (*1*). The symptoms arise from altered neuronal morphology and loss of synaptic connections within the cerebellum (*2*). Treatments for ataxia are primarily physical therapies, as there are currently no pharmacological tools to address the underlying causes of this condition (*3*). Inherited forms of ataxia are often associated with mutations of membrane proteins such as transporters and ion channels (*4*–*6*). Notably, a forward genetic screen that uncovered a dominant mouse model of cerebellar ataxia—the moonwalker mouse—identified a gain-of-function (GOF) mutation (T635A) in the canonical transient receptor potential member 3 (TRPC3) channel (*7*–*9*). However, it is unclear how this single-point mutation accounts for the observed neurodegenerative phenotype, as TRPC3 function appears unaffected in other tissues (*7*, *9*).

TRPC channels are a family of tetrameric, nonselective cation channels activated by intracellular signaling molecules. TRPC1/4/5 channels are activated by Gα_i/o_ heterotrimeric guanine nucleotide–binding proteins (G proteins) (*10*), whereas TRPC3/6/7 channels are activated by diacylglycerol (DAG) (*11*, *12*); TRPC2 is a pseudogene in humans (*13*). Although expressed throughout the body (*14*–*17*), TRPC3 is particularly abundant in Purkinje neurons and unipolar brush cells in the cerebellum (*8*, *18*), where it regulates the excitability of an essential circuit for motor control and coordination (*19*). Within the cerebellum, TRPC3 undergoes alternative splicing that produces an isoform lacking 28 amino acids from the intracellular C-terminal loop linking the TRP and rib helices (isoform c; Δ28) (*20*, *21*). We have demonstrated that this splice variant exhibits an altered response to the TRPC3 agonist GSK1702934A (GSK170) when transiently expressed in human embryonic kidney (HEK) 293 cells (*22*). Specifically, the shortened C-terminal loop in the Δ28 splice variant increases channel activity, whereas elongating the loop has the opposite effect, suggesting that there may be allosteric coupling between the cytoplasmic and transmembrane domains (*22*). The missing 28 amino acids encompass a part of the putative calmodulin/inositol 1,4,5-trisphosphate receptor–binding site, which may relieve the channel from inhibition by calmodulin (*20*, *23*–*25*). Nonetheless, it remains to be determined how this naturally occurring TRPC3 splice isoform contributes to the onset of cerebellar neurodegeneration and other neurological disorders.

Beyond the cerebellum, dysregulated TRPC3 activity contributes to vascular constriction and maladaptive cardiac remodeling, promotes pancreatitis, and drives fibrosis and the progression of select cancers (e.g., gastric and ovarian) (*26*). These roles underscore the need to define its structure across functional states and to identify druggable regulatory sites. For instance, TRPC3 structures determined in the presence of various agonists have not yet revealed the open-state conformation (*27*, *28*). Furthermore, there are no TRPC3 inhibitors in clinical use, as existing ones have poor metabolic stability (*29*), requiring the development of additional drugs. The absence of structural data for TRPC3 in active or inhibited conformations has hindered efforts to develop allosteric modulators, an increasingly promising strategy for achieving subtype-selective and physiologically relevant inhibition (*30*).

To understand why a single-point mutation in TRPC3 increases susceptibility in cerebellar neurons, we used a multimodal approach that combined electrophysiological assays, in vivo neurodegeneration models, high-resolution cryo–electron microscopy (cryo-EM), and computational analyses. Here, we describe a mechanism whereby a combination of a pathogenic mutation and a hyperactive splice isoform stabilizes the channel’s open state. Using cryo-EM and molecular dynamics (MD) simulations, we present the structure of TRPC3 in an open state and identify an allosteric inhibitory site. These findings may help to explain why cerebellar neurons are particularly vulnerable in TRPC3-associated ataxias.

## RESULTS

### An amino acid triad regulates TRPC3 function

**Fig. 1.**
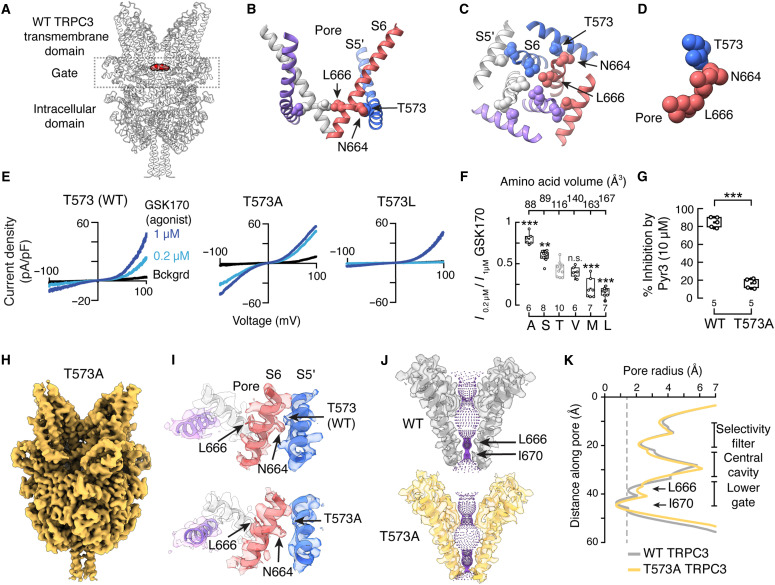
The T573A mutation increases TRPC3 function and reduces gate hydrophobicity. (**A**) Side view of the wild-type (WT) hTRPC3 tetramer structure [Protein Data Bank (PDB) 9OLK], highlighting L666 in red. (**B**) Expanded side view of the S5′/S6 bundle crossing depicting interactions between the T573, N664, and L666 triad. Each subunit is shown in a different color. (**C** and **D**) Top views of the hTRPC3 amino acid triad. (**E**) Representative current-voltage relationships determined by whole-cell patch-clamp recordings of HEK293 cells expressing WT hTRPC3, or T573A and T573L mutants in the absence (Bckgrd) and presence of 0.2 (pale blue) and 1 μM (saturating concentration, blue) GSK1702934A (GSK170, a TRPC3 agonist). (**F**) Boxplots showing mean (square), 75th to 25th percentiles (box boundaries), and outlier range with 1.5 coefficient (whiskers) for 0.2 μM/1 μM GSK170 currents through T573 hTRPC3 mutant channels at 100 mV. T573 mutants plotted by amino acid volume. One-way analysis of variance (ANOVA) (*F* = 55.12, *P* = 4 × 10^−16^) with Tukey multiple-comparisons test. *n* is indicated below each box. (**G**) Boxplots of the percentage of WT and T573A TRPC3 currents elicited by GSK170 (1 μM) and inhibited by Pyr3 (10 μM). Currents were obtained by whole-cell patch-clamp recordings (+100 mV). Two-sample *t* test (*t* statistic = 19.8, *P* = 4.4 × 10^−8^). (**H**) Cryo-EM map of the T573A mutant (EMD-70596). (**I**) Expanded side view of S5′/S6 bundle crossing highlighting the increased size of the interhelix cavity between T573A (S5′) and N664 (S6). (**J**) Permeation pathway of WT and T573A hTRPC3. (**K**) Pore profiles of WT and T573A hTRPC3. L666 and I670 are indicated by arrows. The dashed gray line indicates a 1.4-Å radius. ***P < 0.001 and **P < 0.01. n.s., not significant.

Although previous work has shown that the GOF TRPC3 moonwalker mutation (T635A) causes cerebellar neurodegeneration (*7*, *9*, *31*), the underlying mechanism remains unresolved (*32*). T635 is highly conserved across a wide range of TRPC3 orthologs (fig. S1A), including human TRPC3 (hTRPC3), for which the equivalent position is T573. This residue is located at the lower region of the S5 helix, where it forms a structural triad with N664 and L666 on the lower half of the S6 helix of the adjacent subunit in the tetramer at the hydrophobic constriction of the pore—the lower gate (Fig. 1, A to D). This triad (T-N-L) is identical in the TRPC family members activated by DAG, suggesting a conserved gating mechanism (fig. S1B). The location of this amino acid triad indicates that it plays a crucial role in stabilizing the closed state of TRPC3; thus, its disruption could facilitate channel opening. The T573A mutant exhibits increased activity compared with the wild-type (WT) channel, as demonstrated by macroscopic current analyses of HEK293 cells transiently expressing hTRPC3 and challenged with GSK1702934A (GSK170) (Fig. 1, E and F). Moreover, the alanine substitution disrupts TRPC3 rectification, and it behaves more like a simple ohmic conductor (Fig. 1E). An amino acid residue scan at T573 revealed a negative correlation [correlation coefficient (*r*) = −0.89] between TRPC3 currents and amino acid volume, showing that small-volume side chains at this position enhance channel activity, likely by relieving the steric constraint of the amino acid triad (Fig. 1, E and F, and fig. S1, C and D). We also measured the effect of Pyr3—a pyrazole compound that selectively inhibits TRPC3 and has been shown to alleviate features of TRPC3-related diseases (*33*–*35*). Unexpectedly, Pyr3 failed to inhibit currents through the T573A mutant, highlighting the impact of the alanine substitution on TRPC3 (Fig. 1G). These findings underscore the need for next-generation inhibitors that selectively target hyperactive TRPC3 variants implicated in neurodegeneration.

To understand the impact of the T573A mutation on the protein structure and its potential effect, we solved the structures of WT hTRPC3 and the T573A mutant at 2.8- and 3.1-Å resolutions, respectively, using single-particle cryo-EM (Fig. 1, A and H; figs. S2 and S3; and tables S1 and S2). As expected, the alanine substitution lacks the interaction with residue N664, increasing the dimensions of the interhelix cavity (S5′ and S6 helices; Fig. 1I). The enlarged cavity likely favors S6 movement and expansion of the permeation pathway upon agonist activation. Consistent with this idea, we observed that L666 (located above the narrowest constriction of the permeation pathway, I670) slightly rotated away from the central pore axis in the apo state compared to WT (Fig. 1, J and K). However, the constriction at I670, formed by the crossing of the four S6 helices, is compatible with a closed state. The movement of L666 decreases the hydrophobicity of the lower gate (fig. S2G), which could account for the enhanced function of the T573A mutant upon agonist activation. These data support a model in which the T573A mutation increases TRPC3 activity by disrupting a critical amino acid triad, thereby relieving steric constraints that stabilize the hydrophobic lower gate in the closed state.

### A cerebellar splice variant and GOF mutation stabilize the TRPC3 open state

**Fig. 2.**
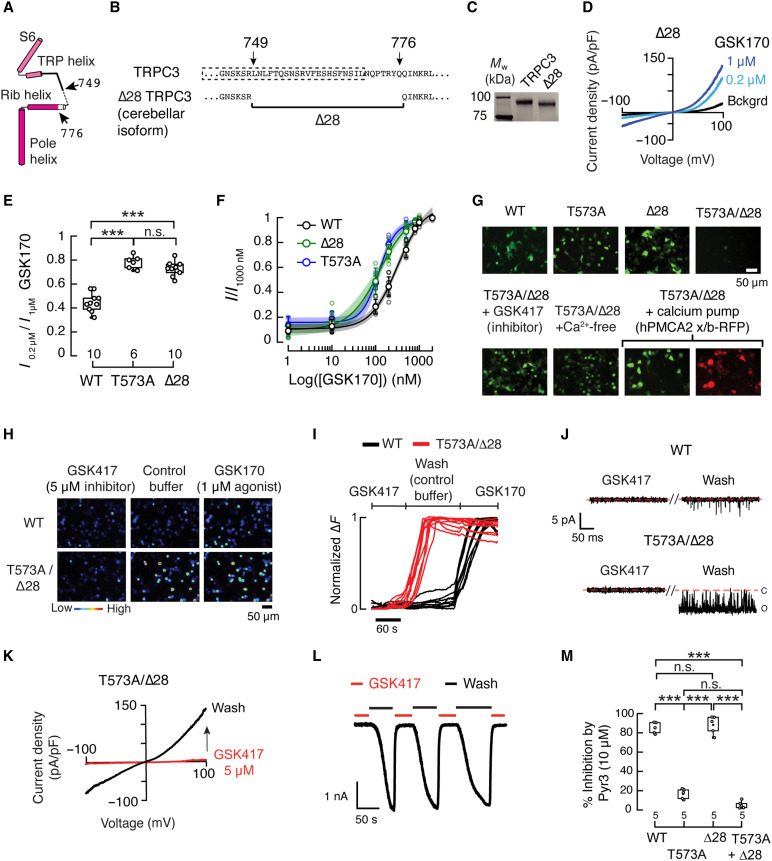
Combined T573A and Δ28 render TRPC3 constitutively open. (**A**) Cartoon and (**B**) sequence changes that generate hTRPC3 Δ28. (**C**) Stain-free gel of purified hTRPC3: WT (97 kDa) and Δ28 (94 kDa). *M*_w_, relative molecular weight. (**D**) Current-voltage relationships from whole-cell patch-clamp recordings of HEK293 cells expressing Δ28 hTRPC3 in the absence (Bckgrd) and presence of 0.2 (pale blue) and 1 μM (blue) GSK170 (agonist). (**E**) Boxplots showing mean (square), 75th to 25th percentiles (box boundaries), and outlier range with 1.5 coefficient (whiskers), for 0.2 μM/1 μM GSK170 currents through various hTRPC3 constructs at 100 mV. One-way ANOVA (*F* = 65.95, *P* = 2.98 × 10^−10^) with Bonferroni multiple-comparisons test. (**F**) Normalized current GSK170 dose-response profiles of various hTRPC3 constructs. A Boltzmann function was fitted to the data. Shaded areas indicate 95% confidence bands. Enlarged circles are means ± SD. (**G**) Micrographs of HEK293 cells expressing green fluorescent protein (GFP) and various hTRPC3 constructs in different conditions: GSK417651A (GSK417, a TRPC3 inhibitor), Ca^2+^-free media, and cotransfection with hPMCA2. Scale bar, 50 μm. (**H**) Micrographs of HEK293 cells expressing WT and T573A/Δ28 hTRPC3 in various conditions, analyzed for their responses using Ca^2+^ imaging; color bar indicates relative change in fluorescence intensity. Scale bar, 50 μm. (**I**) Traces of normalized (Δ*F*) intensity changes from (H). Scale bar, 60 s. (**J**) Single-channel recordings of WT and T573A/Δ28 hTRPC3 in the presence and after washout of GSK417. (**K**) Current-voltage relationships of HEK293 cells expressing T573A/Δ28 in the presence (red) and after washout (black) of GSK417. (**L**) Currents through T573A/Δ28 at −60 mV in the same conditions as (K). Scale bar, 1 nA/50 s. (**M**) Boxplots showing inhibition of GSK170-induced currents (1 μM) by Pyr3 (10 μM) at 100 mV. One-way ANOVA (*F* = 238.27, *P* = 1.7 × 10^−13^) with Bonferroni multiple-comparisons test. ***P < 0.001.

Because TRPC3 is expressed in various tissues throughout the body (*14*–*17*), it is unclear why the pathological changes associated with the GOF mutation appear to be largely confined to the cerebellum (*7*). Within the hindbrain, TRPC3 undergoes alternative splicing (*20*, *21*), resulting in a shorter C-terminal loop (Δ28; Fig. 2, A to C). Similar to the T573A mutant, this cerebellar-specific TRPC3 isoform (c; Δ28) features enhanced activity in response to direct agonist activation (Fig. 2, D to F). To mimic mutant channels within the cerebellum, we introduced the T573A mutation on the Δ28 TRPC3 isoform (T573A/Δ28). Notably, HEK293 cells transiently transfected with T573A/Δ28 failed to survive in the culture media, unlike cells transfected with only T573A or Δ28 TRPC3 (Fig. 2G). Incubation with the TRPC3 inhibitor GSK417651A (GSK417), or removal of extracellular Ca^2+^, substantially improved the survival of cells expressing the T573A/Δ28 hypermorphic variant.

To test whether TRPC3-mediated Ca^2+^ influx was enhanced by T573A/Δ28, we measured fluorescence changes using Fluo-4. HEK293 cells were transfected with T573A/Δ28 in the presence of GSK417, and fluorescence intensity was measured upon washout. In the absence of the agonist, we observed an increase in intracellular Ca^2+^ in T573A/Δ28-transfected cells but not in WT-transfected cells (Fig. 2, H and I). We speculated that this baseline Ca^2+^ influx might underlie the reduced viability of cells expressing T573A/Δ28 and that adding an inhibitor or reducing intracellular Ca^2+^ might reduce cell death. We tested this hypothesis by using the human plasma membrane calcium pump isoform 2 (hPMCA2), which extrudes Ca^2+^ from cells and has overlapping expression with TRPC3 in the brain (*36*, *37*). Coexpression of hPMCA2 with the T573A/Δ28 variant substantially enhanced cell survival compared with expression of T573A/Δ28 alone (Fig. 2G), underscoring the therapeutic potential of calcium pump activation in mitigating TRPC3-mediated Ca^2+^ overload.

We then performed single-channel and macroscopic recordings of HEK293 cells transiently transfected with T573A/Δ28 in the presence of the inhibitor GSK417. Functional analyses demonstrated that the T573A/Δ28 variant results in a constitutively open TRPC3 channel (Fig. 2, J to L, and fig. S4, A and B). Upon washout of the inhibitor, we observed that the T573A/Δ28 mutant remained open at voltages equivalent to the resting potential of neurons (−60 mV; Fig. 2, K and L). Likewise, combining T635A and Δ28 in the TRPC3 mouse ortholog also results in a constitutively open channel (fig. S4, C and D). These findings suggest that the absence of 28 amino acids from the intracellular C-terminal loop could facilitate conformational changes at the lower gate (i.e., I670). Together with the L666 rotation observed in the T573A mutant, these changes may further reduce hydrophobicity and stabilize the open state. The lead TRPC3 inhibitor Pyr3 also fails to inhibit the T573A/Δ28 (Fig. 2M). Our findings demonstrate that the combination of a GOF mutation and a cerebellum-specific splice variant yields a constitutively open TRPC3 channel, providing a potential mechanistic explanation for the tissue-specific susceptibility observed in TRPC3-associated ataxias. These results also highlight the need for selective inhibitors that can effectively target hypermorphic TRPC3 variants.

### A hypermorphic TRPC3 variant causes neurodegeneration in worms

**Fig. 3.**
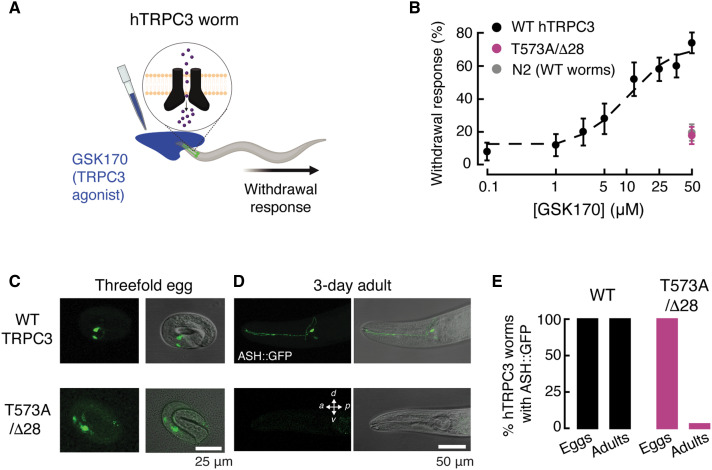
Constitutively open TRPC3 promotes neurodegeneration in worms. (**A**) Cartoon of GSK170 drop worm assay [Created in BioRender. Vásquez, V. (2026) https://BioRender.com/dpjd1xk]. (**B**) GSK170 dose-response profile for WT (N2) worms (gray) and worms expressing WT (black) and T573A/Δ28 (magenta) hTRPC3. *n* ≥ 25 worms per condition. (**C** and **D**) Fluorescent and merged bright-field micrographs of ASH::GFP neurons in eggs and adults of WT and T573A/Δ28 hTRPC3-expressing strains. Scale bar, 25 μm in (C) and 50 μm in (D). (**E**) Percentage of worms expressing GFP in ASH neurons of WT and T573A/Δ28 hTRPC3-expressing strains (*n* = 95 and 102, respectively).

Neurodegenerative diseases are characterized by a profound loss of neuronal density and function, resulting in memory loss and ataxia (*38*, *39*). Such pathological phenotypes can be investigated in the model organism *Caenorhabditis elegans* (*40*–*42*). For example, a GOF mutation in the ion channel DEG-1, which is expressed in a pair of *C. elegans* sensory neurons known as ASH, has been found to cause late-onset neuronal degeneration (*43*). We exploited the multimodal nociceptive function of ASH neurons (*44*, *45*) and the absence of TRPC3 in the worm genome to investigate the consequences of constitutively open TRPC3 channels. We generated integrated transgenic strains expressing WT or T573A/Δ28 TRPC3 in ASH neurons carrying green fluorescent protein (GFP) and then performed behavioral tests on moving adult worms by placing a drop of the agonist GSK170 in front of them (Fig. 3, A and B). GSK170 induced a dose-dependent withdrawal behavior in worms expressing WT TRPC3 but not those expressing T573A/Δ28 or WT worms (N2; Fig. 3B). Furthermore, imaging experiments showed that worms expressing T573A/Δ28 TRPC3 had ASH neurons during embryonic stages but not at adulthood (Fig. 3, C to E). These results mirror the neurodegeneration observed in the mouse model of cerebellar ataxia and demonstrate that the T573A/Δ28 variant induces posthatching degeneration of ASH neurons. They also establish this *C. elegans* model as a tractable system for investigating TRPC3 physiology and pharmacological targeting in vivo.

### The TRPC3 pathogenic variant has a wide pore

**Fig. 4.**
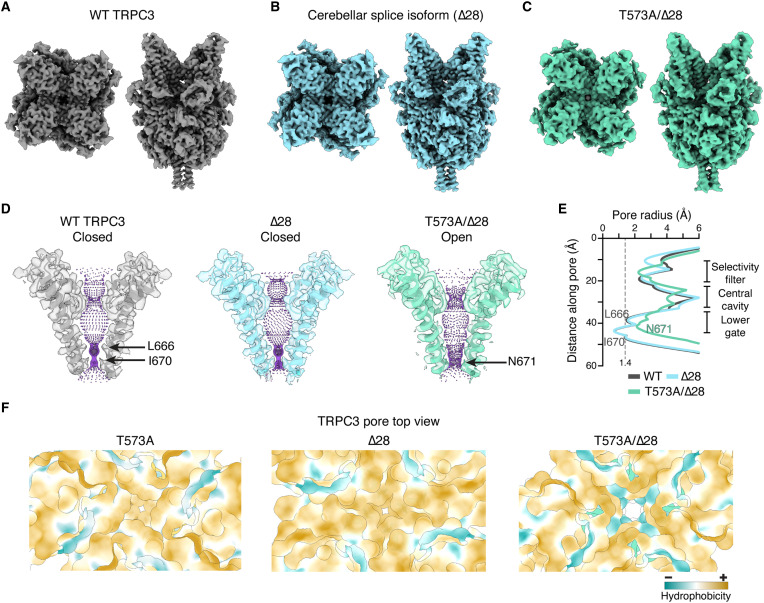
Expansion of the TRPC3 permeation pathway. (**A** to **C**) Top- and side-view cryo-EM maps of WT (EMD-70595), Δ28 cerebellar isoform (EMD-70597), and T573A/Δ28 (EMDB-70724) hTRPC3 constructs. (**D** and **E**) Permeation pathways and pore profiles of WT (closed state, PDB 9OLK), Δ28 (closed state, PDB 9OLM), and T573A/Δ28 (open state, PDB 9OPU) hTRPC3. The dashed gray line indicates a 1.4-Å radius. See also figs. S5 and S6. (**F**) Cross section through the pore showing lipophilic surface potential of T573A, Δ28, and T573A/Δ28. Surfaces are colored by relative hydrophobicity from dark goldenrod (most hydrophobic) to dark cyan (most hydrophilic).

The closed-state structure of WT hTRPC3 has been solved in detergent and nanodiscs (*22*, *27*, *28*, *46*, *47*). To determine the structural impact of the cerebellum-specific splice variant (Δ28), we analyzed this construct in digitonin using single-particle cryo-EM and compared it with the WT (Fig. 4, A and B, and figs. S3 and S5). We determined the structure of the Δ28 at 2.5-Å resolution. The architecture mirrored the overall structure and pore profile of the full-length channel, except for the missing residues in the C-terminal loop that connects the TRP and rib helices (Fig. 4, D and E; fig. S5, A to C; and tables S1 and S2).

The structural changes observed in either the T573A mutant or the Δ28 splice variant could not explain the robust stabilization of the open state that we measured in functional and in vivo experiments. Therefore, we determined the structure of T573A/Δ28 at 3.3-Å resolution (Fig. 4C and fig. S6). The hydrophobic-gate residues (L666 and I670) rotate away from the pore axis, dilating the permeation pathway and widening the opening at the S6 bundle crossing. This produces a markedly different pore profile and reduces hydrophobicity at the lower gate compared with full-length, T573A, or Δ28 TRPC3 structures (Fig. 4, D to F, and movie S1). The expansion observed in our structure resembles that seen in previously resolved TRP channels in the open state (*48*–*52*). Notably, we were unable to identify a class representing the closed state in the T573A/Δ28 dataset. We therefore conclude that this expanded architecture represents the structure of an open TRPC3 channel. This structural insight provides a framework for the rational design of novel inhibitors targeting this pathogenic TRPC3 variant and potentially other hypermorphic TRPC family members.

### Conformational changes associated with TRPC3 opening

**Fig. 5.**
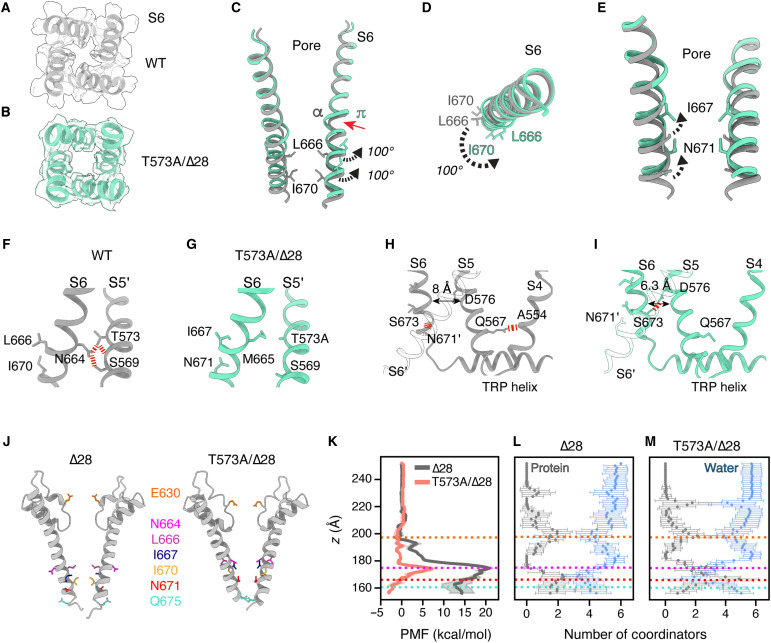
Opening of TRPC3 lower gate. (**A** and **B**) Top-view density map of WT and T573A/Δ28 hTRPC3. (**C**) Side-view superposition of S6 helices from two opposing subunits of WT and T573A/Δ28 hTRPC3. α-to-π helical transition shown by red arrow, rotation of hydrophobic gate residues L666 (~100°) and I670 (~100°) shown by black arrows. (**D**) Top-view superposition of S6 helices depicting rotation of hydrophobic residues away from the permeation pathway. (**E**) Side-view superposition of S6 helices from two opposing subunits. Rotation of I667 and N671 toward the permeation pathway is shown by black arrows. (**F** and **G**) Expanded side view of S6/S5′ bundle crossing showing hydrogen bond interactions between T573, N664, and S569 in WT but not in T573A/Δ28 hTRPC3. (**H** and **I**) Expanded side view of bundle crossing showing hydrogen bond interactions between the S4, S5, and S6 helices in WT hTRPC3 and T573A/Δ28. See also fig. S7. (**J**) Key residues along the ion permeation pathway comprising the selectivity filter and hydrophobic gate in the closed and open states. (**K**) Na^+^ PMF in ∆28 and T573A/∆28 computed from umbrella sampling in MD simulations. The horizontal dashed lines indicate locations of residues highlighted in (J) that are primarily involved in coordinating Na^+^ ions in the permeation pathway. (**L** and **M**) Inner shell coordination structure of Na^+^ in the permeation pathway for ∆28 (L) and T573A/∆28 (M). The numbers of water and protein coordinators are plotted separately, and error bars represent fluctuations around averages.

The pore diameter of T573A/Δ28 increases after a counterclockwise rotation of L666 and I670 (~100°) at the S6 bundle crossing, consistent with a reduced hydrophobic barrier, and is accompanied by α-to-π helical transition of the S6 helix (Fig. 5, A to D). This α-to-π helical transition has not yet been observed in other TRPC subfamily members in the context of channel opening but is a hallmark of activation in other TRP channels (*53*, *54*). As a result of the S6 helix rotation, residues I667 and N671 are now oriented toward the permeation pathway (Fig. 5E). Although I667 slightly faces the pore, its positioning does not create a constriction like L666 or I670 in the other channel structures (Fig. 4, D and E). In other TRP channels, a pore-facing asparagine residue has been proposed to increase hydration of the permeation pathway, thereby promoting ion conduction (*55*–*57*). Similarly, the polar residue N671 in TRPC3 may enhance local hydration, support ion coordination, and facilitate ion permeation through the pore.

In the full-length WT structure, the amino acid triad at the lower gate (T573, N664, and L666) stabilizes the closed state. Analysis of hydrogen bonds between S6 and S5′ reveals that the interactions between T573 and N664, N664 and S569, and T573 with the backbone of S569 are disrupted in the T573A/Δ28 structure (Fig. 5, F and G). In the open state, M665 rotates to the position previously occupied by N664 in the closed state but does not reestablish the hydrogen bonds. Our results support the notion that the T573A mutation contributes to the helical rotation of S6 in the T573A/Δ28 variant by disrupting these hydrogen bonds. Gating of WT TRPC3 may thus involve the dynamic disruption and reorganization of this network of interactions. Because the amino acid triad and associated hydrogen bonds are conserved among DAG-activated TRPC channels, our results support a common mechanism of channel activation across the TRPC3/6/7 subfamily (fig. S1B).

Stabilization of the S6 helix in the open state occurs through molecular rearrangements at the channel bundle crossing. Disruption of the interactions between S673 (S6) and N671 (S6′) at the base of the S6 helix and between Q567 (S5) and the backbone of A554 (S4) releases the constraints of the S6 and S5 helices (Fig. 5, H and I). Together with the rotation of the S6, a new interaction forms between S673 (S6) and D576 (S5), bringing the S6 and S5 helices closer (from 8 to 6.3 Å). Furthermore, we also observed another bond between N655 (S6) and S624 (pore helix) that stabilizes the new conformation of S6 and S5 helices, as well as the π helix (fig. S7). Together, these rearrangements serve as anchors that stabilize the channel in its open state. Our previous functional studies suggest that there is allosteric coupling between the cytoplasmic and transmembrane domains, as shortening the length of the C-terminal loop increases TRPC3 activity, while elongating it has the opposite effect (*22*). We propose that the shortened C-terminal loop (Δ28) stabilizes the new conformation of the S5 and S6 helices, including the S6 bundle crossing, observed in the T573A/Δ28 structure.

Because of S6 rotation, the residues that form the hydrophobic plug (L666 and I670) shift away from the permeation pathway, while I667, another hydrophobic residue, reorients toward the pore. To assess whether I667 or other lower gate residues create an energetic barrier to ion translocation, we calculated the potential of mean force (PMF) for Na^+^ ions permeating through the Δ28 and T573A/Δ28 structures using MD simulations. We find that the translocation barrier in T573A/Δ28 is substantially lower compared with the Δ28 structure (Fig. 5, J to M). During permeation in T573A/Δ28, we observe that Na^+^ primarily passes through the pore coordinated by water molecules, although in certain regions, it also interacts directly with amino acid side chains. At the selectivity filter, ions are stabilized by the carboxylate group of E630. At the lower gate, coordination occurs as expected through the carbonyls of N664 and N671, while below the gate, Q675 provides additional carboxylate coordination. Together, our data support a mechanism in which a low-energy barrier at the hydrophobic gate drives the variant’s hypermorphic activity. Collectively, these findings reveal that new interactions between S6 and the S5 helices facilitate channel opening by widening the lower gate, diminishing the hydrophobic barrier, and stabilizing the open state.

### Inhibition of the TRPC3 open state

**Fig. 6.**
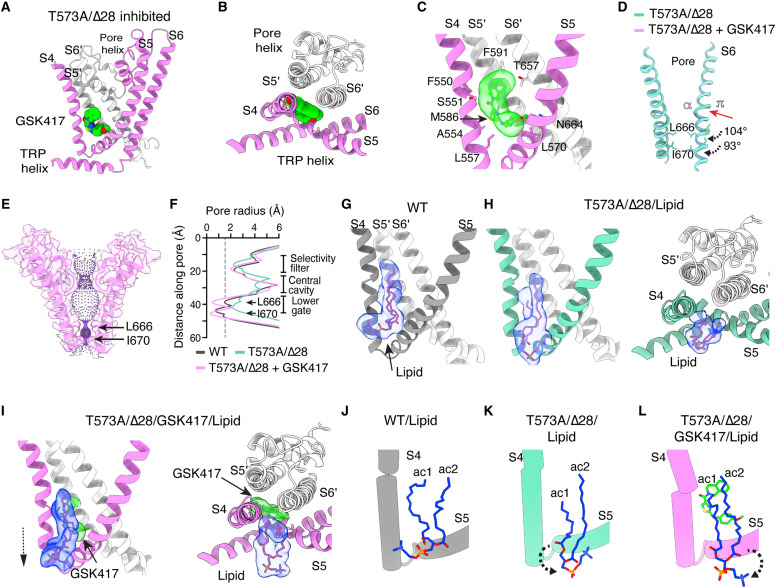
TRPC3 inhibitor stabilizes the closed state of T573A/Δ28. (**A**) Ribbon representation of T573A/Δ28 hTRPC3 (PDB 9OLX) showing the position of GSK417 (green) within a hydrophobic pocket at the subunit interface created by the S4, S5, S5′, S6, and S6′ helices. (**B** and **C**) Expanded top and side views of GSK417 bound to T573A/Δ28. (**D**) Side-view superposition of S6 helices from two opposing subunits of T573A/Δ28 with and without GSK417. π-to-α helical transition shown by red arrow and rotation of hydrophobic gate residues L666 (~100°) and I670 (~100°) toward the pore shown by black arrows. (**E** and **F**) Permeation pathway and pore profile of WT, T573A/Δ28, and T573A/Δ28/GSK417. L666 and I670 are indicated by black arrows. The dashed gray line indicates a 1.4-Å radius. (**G** to **I**) Expanded side and top views of WT, T573A/Δ28, and T573A/Δ28/GSK417 showing the position of a lipid bound within a hydrophobic pocket at the subunit interface created by the S4, S5, S5′, S6, and S6′ helices. (**J** to **L**) Cylinder representation of S4 and S5 helices of WT, T573A/Δ28, and T573A/Δ28/GSK417, highlighting the lipid and lipid/GSK417 complex, respectively, bound in the hydrophobic pocket. See also figs. S8 and S9.

Because our functional experiments highlighted an unexpected resistance of T573A and T573A/Δ28 mutants to Pyr3 inhibition (Fig. 2M) and encouraged by the ability of GSK417 to improve the viability of cells expressing the pathogenic mutant, we sought to determine the structure of T573A/Δ28 in complex with this inhibitor. Depicting the inhibitor binding site could aid the development of more effective treatments for cerebellar ataxia. Accordingly, we determined the structure of GSK417 bound to the T573A/Δ28 variant using single-particle cryo-EM at a resolution of 3.3 Å (figs. S8A and S9). We observed a density consistent with GSK417 in a hydrophobic pocket at the subunit interface formed by S4, S5, S5′, and S6′, which was absent in the T573A/Δ28 open structure (Fig. 6, A to C, and fig. S8B). GSK417 induced a clockwise rotation of the S6 helix, resulting in its transition from a π to α helix and reorientation of the hydrophobic residues L666 and I670 toward the permeation pathway (Fig. 6D and fig. S8C). We also observed that the rotation of the helix restores the interaction between Q567 and the backbone of A554, present in the closed state (fig. S8D). The permeation pathway and pore profile of the inhibited state indicate that L666 and I670 provide a greater constriction of the pore than in the closed WT channel (Fig. 6, E and F). As expected, there was an increase in the pore hydrophobicity compared with the T573A/Δ28 open structure in the absence of the inhibitor (fig. S8E).

In the transient receptor potential vanilloid 1 (TRPV1), a phosphatidylinositide lipid binds to the vanilloid pocket (*58*). Likewise, we observed a phospholipid occupying the hydrophobic pocket in our WT structure and T573A/Δ28 with and without GSK417 (Fig. 6, G to L). In the T573A/Δ28 mutant, the lipid headgroup and acyl chain reorient within the hydrophobic pocket compared with the WT (Fig. 6, J and K). The reorganization of the lipid may also reduce the steric constraints on the S5 and S6 helices, favoring channel opening. In the inhibited state, the GSK417 fills the position previously occupied by one of the phospholipid acyl chains (ac1) in the closed channel and reorients the lipid head group downward (Fig. 6, I and L). These data support that a phospholipid or inhibitor in the hydrophobic pocket stabilizes the nonconductive states, while the conformational changes associated with channel opening alter phospholipid coordination. The structure of the constitutively open TRPC3 mutant bound to an inhibitor reveals the therapeutic potential of targeting a conserved hydrophobic pocket to counteract GOF mutations in TRPC3 and potentially other TRPC subfamily members.

## DISCUSSION

Cerebellar ataxias result from the dysfunction or degeneration of Purkinje neurons and are characterized by impaired motor coordination (*1*). Understanding the molecular mechanisms that lead to neurodegenerative disorders is crucial for developing effective treatment strategies. Here, we used a multimodal approach that integrates electrophysiological assays, in vivo neurodegeneration models, high-resolution cryo-EM, and MD simulations to uncover how a hypermorphic variant leads to localized neurodegeneration. Our study demonstrates that combining the cerebellar TRPC3 isoform, which lacks 28 residues, with the T573A mutation results in constitutive channel activation, leading to cell death in culture and neurodegeneration in *C. elegans*. This variant exhibits a distinct pharmacological profile, emphasizing the significance of targeting disease-relevant pathogenic channels in drug discovery. Notably, we demonstrate that the coexpression of a neuronal calcium pump alleviates TRPC3-mediated Ca^2+^ toxicity, suggesting alternative therapeutic strategies for this condition. Cryo-EM structures of the open and inhibitor-bound states of the variant reveal conformational changes that promote the open state, highlighting a hydrophobic pocket that stabilizes the closed conformation and offering mechanistic insight into ion channel regulation. MD simulations support a reduction in the barrier height at the hydrophobic gate.

**Fig. 7.**
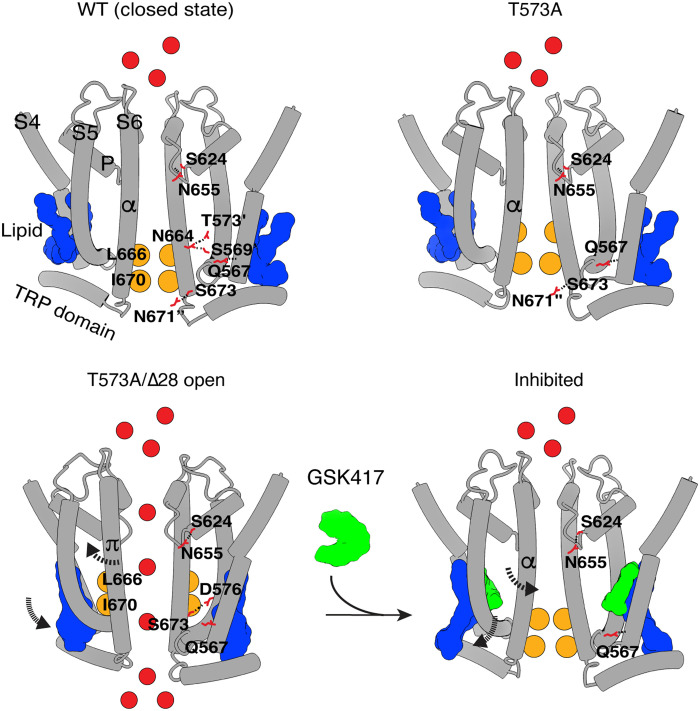
Model of TRPC3 opening and inhibition. The cartoon summarizes the key structural changes that emerged from our functional and structural analysis. Comparison between the closed, open, and inhibited states.

Figure 7 and the supplementary movie summarize the key structural changes that emerged from our functional and structural analyses. A comparison between the closed, open, and inhibited conformations reveals key mechanistic highlights of gating: The amino acid triad and I670 at the gate of WT hTRPC3 maintain a nonconductive pore, stabilized by a phospholipid in the hydrophobic pocket. The T573A mutation disrupts the triad, enabling L666 to rotate away from the central axis to reduce hydrophobicity and facilitate pore opening. The T573A mutation decreases TRPC3 rectification, likely by altering pore geometry/accessibility and thereby facilitating inward current. Alternatively, the mutation could reduce ion- or small molecule–induced channel block. Widening of the pore in T573A/Δ28 is accompanied by an α-to-π helical transition of the S6, reorganization of the hydrogen-bonding network, and reorientation of key side chains, facilitating ion conduction and lowering the energy barrier at the hydrophobic gate. The open state is further stabilized by a reorientation of the phospholipid in the hydrophobic pocket. A shorter C-terminal loop might also stabilize the reduced distance between S6 and S5, promoting the open state. However, since this region has not been fully resolved in any member of the TRPC channel family (*25*), including our structures, it is challenging to draw mechanistic conclusions at this time. In the inhibited state, S6 of the constitutively open mutant transitions back to an α helix, positioning the hydrophobic gate residues back toward the permeation pathway. In this nonconductive state, the hydrogen bonds resemble those in the WT closed state, and the hydrophobic pocket is occupied by an inhibitor (GSK417) and a phospholipid. Together, we unveil the structural features of TRPC3 that could contribute to neurological disorders and identify a previously uncharacterized druggable inhibitory binding site.

Alternative splicing enhances proteome diversity and plays a pivotal role in regulating ion channel activity (*59*, *60*), particularly through tissue-specific modulation of their function. Many TRP channels have splice variants that show impaired trafficking or reduced activity. In contrast, cases where splicing enhances channel function are less common (*59*). A notable example is the alternative splicing of TRPV1 transcripts in the trigeminal ganglia of vampire bats, yielding a channel with a shorter cytoplasmic domain that is easier to open (i.e., reduced threshold for thermal activation) (*61*). Similar to TRPV1, our study underscores the physiological significance of a brain-specific TRP channel isoform, demonstrating that splicing-mediated enhancement of channel activity extends to other TRP family members. The contribution of these isoforms, alone or in combination with gain- or loss-of-function mutations, to disease progression has been underestimated and merits further investigation.

Increased activity of TRPC3 has been linked to various neurological disorders, including cerebellar ataxia, aging-related dysfunction, and Alzheimer’s disease (*62*–*66*), where excessive calcium influx may originate from various sources, including GOF mutations, alternative splicing, or a combination. Beyond the ataxic mutant, elevated expression of the overactive splice isoform may promote cytotoxic calcium overload, leading to neuronal degeneration. Increasing the activity of calcium pumps or identifying novel molecules that target the TRPC3 inhibitory hydrophobic pocket could help counteract the heightened activity of WT TRPC3 or hypermorphic variants in the context of neurological disorders. Our study shows that the cerebellum-specific splice variant of TRPC3 may underlie the tissue-specific motor-centered disorder, reveals the structural features of TRPC3 that contribute to neurological disorders, and lays the groundwork for developing effective pharmacological therapies.

## MATERIALS AND METHODS

### Channel constructs

For electrophysiology and imaging experiments, human and mouse TRPC3 constructs, as well as GFP, were cloned into a pMO vector (a pcDNA3.1-based vector with the 5′ and 3′ untranslated regions of the β-globin gene; Epoch Life Science). For protein purification experiments, hTRPC3 constructs were cloned into a BacMam vector, as previously described (*67*). Briefly, an N-terminal fusion cassette containing the Kozak sequence, maltose-binding protein (MBP), and a tobacco etch virus (TEV) protease site was inserted between the mammalian cytomegalovirus promoter and the protein gene to facilitate purification using amylose resin.

### Cell transfections

We used Lipofectamine 2000 (Thermo Fisher Scientific) according to the manufacturer’s instructions. HEK293 (American Type Culture Collection) cells were grown in six-well plates to 60 to 70% confluency and cotransfected with GFP (0.1 μg/ml) and TRPC3 (1.1 μg/ml) constructs for macroscopic currents and fluorescence imaging. For single-channel recordings, HEK293 cells were cotransfected with GFP-pMO (0.05 μg/ml) and TRPC3 constructs (0.15 μg/ml). For the constitutively open construct, the culture media was supplemented with 5 μM TRPC3 inhibitor GSK417651A to maintain cell viability. For electrophysiology, all transfected cells were used 18 to 24 hours posttransfection. For fluorescence imaging, all transfected cells were imaged 48 hours posttransfection.

### Cell culture and electrophysiology

HEK293 cells were cultured in Dulbecco’s modified Eagle’s medium (Invitrogen) supplemented with 10% fetal bovine serum and 1% penicillin-streptomycin at 37°C and 5% CO_2_. Macroscopic currents in the whole-cell patch-clamp configuration were recorded 18 to 24 hours posttransfection. The extracellular solution contained 140 mM NaCl, 2.8 mM KCl, 1 mM MgCl_2_, and 2 mM Hepes (pH 7.4). Pipettes were made of borosilicate glass (outer diameter, 1.5 mm; inner diameter, 1.10 mm; Sutter Instruments) and fire polished with a resistance between 2.8 and 4.0 MΩ when filled with an intracellular solution that contained 140 mM CsCl, 5 mM EGTA, and 10 mM Hepes (pH 7.2). Currents were recorded with a Multiclamp 700B amplifier (Molecular Devices) using a 1-s ramp from −100 to 100 mV. TRPC3 channel agonist GSK1702934A and the inhibitor GSK417651A (Focus Biomolecules) were dissolved in dimethyl sulfoxide and freshly dissolved in bath solution to the indicated concentration. Single-channel recordings were done in the outside-out configuration of the patch-clamp technique using a bath solution of 140 mM NaCl, 2.8 mM KCl, 1 mM MgCl_2_, and 2 mM Hepes (pH 7.4) and an intracellular solution that contained 140 mM CsCl, 5 mM EGTA, and 10 mM Hepes (pH 7.2). Outside-out patches were clamped at a constant voltage (−60 mV), and data were acquired using a gap-free protocol. Single-channel current amplitudes were determined by fitting a Gaussian function to all-point histograms generated from 1-min recordings of three independent preparations. Data were acquired with a sampling rate of 20 kHz, low-pass filtered (2 kHz), and analyzed offline using Clampfit v10.4.2.0 (Molecular Devices).

### Calcium imaging

HEK293 cells were cultured, as described above, and loaded with 1 μM Fluo4-AM (Thermo Fisher Scientific), according to the manufacturer’s protocol. Micrographs were acquired in an upright Olympus BX51WI microscope with a 10× water immersion objective (numerical aperture 0.3) and analyzed using CellSens software (Olympus). The bath solution contained 140 mM NaCl, 6 mM KCl, 1 mM MgCl_2_, 2 mM CaCl_2_, 10 mM glucose, and 10 mM Hepes (pH 7.4). Data analysis was performed offline using OriginLab.

### *C. elegans* strains and behavioral assays

Worms were propagated as previously described (*68*). WT (N2) was obtained from the *Caenorhabditis* Genetics Center, which is funded by the National Institutes of Health Office of Research Infrastructure Programs (P40 OD010440). We used the services of InVivo Biosystems to generate transgenic worms. First, we engineered a transgenic worm expressing WT hTRPC3, COP2236 *knuSi854* [*pnu2311 (osm10p::Hs trpc3::tbb-2u in ttTi5605, unc-119(+))] II; unc-119(ed3) III; osm-9(ky10) IV; Posm-10::GFP X*, using the MosSCI method (*69*, *70*). Second, we used CRISPR-Cas9 to mutate residue T573A and delete 28 residues (Δ28, deletion 749 to 776) from the TRPC3 C terminus to build the T573A/Δ28 worm COP2634 *knu1206 [hsTRPC3 (84bp deletion)]* and *knu1197 [hsTRPC3 (T573A)]* in *knuSi854 [osm-10p::HsTRPC3::tbb-2u in ttTi5605, unc-119(+))] II; unc-119(ed3) III; osm-9 (ky10) IV; Posm-10::GFP X]*.

Behavioral assay. We placed worm eggs on plates seeded with OP50 (an *Escherichia coli* uracil auxotroph strain that is used as a food source for worms). Young adult hermaphrodite worms on the plates (produced from seeded eggs after 3 days at 20°C) were rinsed with M13 buffer (30 mM tris-HCl, 100 mM NaCl, and 10 mM KCl pH 7) and transferred to a plate without food, 15 min prior to the drop-behavioral assay. Behavioral trials were performed by placing a drop containing M13 buffer plus 1% (v/v) ethanol, with or without various concentrations of GSK1702934A, in front of a moving young adult hermaphrodite worm as previously described (*71*). Withdrawal responses were scored as a dichotomous variable. At least 15 young adult hermaphrodite worms were tested in the trial each day (blind to genotype), and the results were compared across three trials.

#### In vivo fluorescence imaging

Worms were individually selected, dropped into 15 μl of M9 buffer, and then paralyzed on a glass slide with 2% agarose pads containing 150 mM 2,3-butanedione monoxime. Bright-field imaging and fluorescence imaging were performed on a Zeiss 710 confocal microscope using 20× or 40× objectives. Micrographs were processed using Fiji ImageJ (*72*) to enhance contrast and convert to an appropriate format.

### hTRPC3 expression and purification

HEK293S GnTI^−^ were cultured in FreeStyle 293 Media (Invitrogen), supplemented with 2% fetal bovine serum (Gibco), and maintained at 37°C with 8% CO_2_ and agitation at 120 rpm. Cultures were infected at 2.3 million cells/ml with 1.7 to 3.5% of a P2 baculovirus, produced using the recombinant Baculovirus Expression System (Bac-to-Bac expression system, Invitrogen). The media of cells expressing the TRPC3 mutants (T573A and T573A/∆28) contained 5 μM GSK417651A. After 18 hours, 10 mM sodium butyrate was added to boost protein expression, and the temperature was lowered to 30°C, without CO_2_. Cells were harvested 72 hours postinfection by centrifugation. The pellet was resuspended in a buffer containing 36.5 mM sucrose, 50 mM tris (pH 8), and 4 mM tris 2-carboxyethyl phosphine (TCEP) supplemented with protease inhibitors: 1 mM phenylmethylsulfonyl fluoride, pepstatin (1 μg/ml), aprotinin (3 μg/ml), and leupeptin (3 μg/ml). Cells were lysed using a high-pressure homogenizer (Avestin), and cell debris was collected by low-speed centrifugation (3000*g* for 15 min at 4°C). The supernatant was ultracentrifuged at 125,000*g* for 55 min at 4°C. The resulting pellet was resuspended in buffer A [300 mM NaCl, 50 mM tris, and 4 mM TCEP (pH 8)] with protease inhibitors. The membranes were flash frozen in liquid nitrogen and stored at −80°C until purification.

Membranes were thawed and solubilized with 1% (w/v) digitonin (Goldbio) rotating at 4°C for 3 hours. The detergent-insoluble material was removed by ultracentrifugation at 125,000*g* for 55 min at 4°C. The supernatant was incubated with amylose resin (New England Biolabs) for 2 hours. After incubation, the resin was loaded into a gravity column and washed with 10 column volumes of buffer A, supplemented with 0.1% digitonin. Protein was eluted with the same buffer containing 20 mM maltose. The protein was incubated overnight with TEV protease (New England Biolabs) to remove the MBP tag. The cleaved protein was isolated by size exclusion chromatography using a Superose 6 column (Cytiva), equilibrated with 200 mM NaCl, 50 mM tris, 4 mM TCEP, and 0.1% digitonin (pH 8). Purified proteins were concentrated to 0.2 to 0.4 mg/ml using an Amicon Ultra-100,000 MWCO filter (Millipore). To determine the structure of the T573A/∆28 + GSK417 complex, we maintained the inhibitor during the entire purification process.

### Cryo-EM sample preparation

Purified TRPC3 protein (3 μl) was applied to 200-mesh copper grids coated with a 2-nm continuous carbon layer (R 2/1, Quantifoil Micro Tools GmbH), which were glow discharged for 15 s at 30 mA. The grids were plunge frozen in liquid ethane using a Vitrobot Mark IV (Thermo Fisher Scientific) under 100% humidity at 4°C, with a 10-s wait time, blot force of −7, and 3.5-s blot time. For the T573A/Δ28-inhibitor complex, the sample was incubated with 50 μM GSK417651A for 30 min at 4°C before vitrification and briefly heated to 37°C for 2 min before grid application and blotting to enhance compound binding.

### Cryo-EM data collection and image processing

Cryo-EM data were acquired using a Titan Krios G3 transmission electron microscope (Thermo Fisher Scientific) operating at 300 keV and equipped with a BioQuantum energy filter (Gatan Inc.). Images were recorded in counting mode at a nominal magnification of ×130,000 on a K2 Summit direct electron detector (Gatan Inc.), yielding a calibrated pixel size of 1.08 Å. Data were collected over a defocus range of −0.8 to −2.2 μm. Each movie stack was recorded over 7 s, split into 35 frames, with a total accumulating electron dose of ~50 e^−^/Å^2^. Automated data acquisition was carried out using EPU software (Thermo Fisher Scientific). Detailed acquisition parameters are listed in table S1.

Image processing was performed using CryoSPARC (Structura Biotechnology Inc.). Motion correction and contrast transfer function (CTF) estimation were carried out using Patch Motion and CTF workflows. For Δ28 TRPC3, particles were picked using a template-based approach with a previously resolved TRPC3 map (EMD-30904) (*47*) low-pass filtered to 20 Å. For all other datasets, particle picking was performed using blob picker.

Following two-dimensional (2D) classification and ab initio reconstruction, particles were subjected to multiple rounds of 2D and 3D classification, heterogeneous refinement, and nonuniform refinement. Final map resolutions were estimated using the gold-standard (Fourier shell correlation = 0.143) criterion. Local resolution estimation and orientation diagnostics were also carried out in CryoSPARC.

### Model building refinement and validation

Initial models were built in Coot and refined in Phenix. For the WT TRPC3 model, a previously resolved TRPC3 structure [Protein Data Bank (PDB): 7DXD] (*47*) was rigid-body fit into the density using ChimeraX, followed by real-space refinement and manual adjustment. The Δ28 TRPC3 model was built using the WT structure as a reference. All subsequent models—including T573A, T573A/Δ28, and the GSK417-bound structures—were constructed using previously refined models. Ligands and lipids were identified using pw_ligands.py (*73*), as previously described (*74*), then fit and refined with the protein model in Phenix. Final model validation used EMRinger, MolProbity, and standard map-model metrics. Molecular interfaces were analyzed in ChimeraX, LigPlot+, and HOLE, and visualizations were prepared using ChimeraX.

### Potential of mean force

All methodological details regarding the setup are provided in the molecular dynamics supplementary methods in the Supplementary Text for molecular simulations. Briefly, the tetramers are inserted into preequilibrated patches of phosphatidylcholine bilayers. Each unit cell contains 542 lipids, about 120,000 waters, and a KCl concentration of 150 mM. Each system is subjected to energy minimization followed by MD with harmonic position restraints placed on protein heavy atoms. After the box dimensions have settled, harmonic restraints are gradually removed, and the final snapshot is used to determine PMFs. One-dimensional PMFs of Na^+^ ions along the pore axis (*z* axis) are estimated using umbrella sampling (*75*) and the weighted histogram analysis method (*76*). The starting conformations for umbrella sampling are obtained from steered MD (SMD) in which all backbone atoms are position restrained (*77*). Initially, umbrella centers from SMD are selected at intervals of roughly 1.5 Å, and Na^+^ is harmonically restrained at these centers using a force constant applied only to the z-coordinate. No other atom positions are restrained in umbrella sampling. We use a total of 60 windows for each structure, and each window is simulated for 20 ns. The final 10 ns of each window is used for analysis, and statistical uncertainties are estimated using block averaging.

### Statistical analysis

No statistical method was used to predetermine the sample size. No data were excluded from the analyses. The experiments were not randomized. All attempts at replication were successful. Electrophysiological and behavioral experiments were performed at least three times on different days with different/independent preparations.

Data were plotted using OriginPro (2024; OriginLab Corp.). Sigmoidal fitting was done using OriginPro with the following Boltzmann function

$$f_{\left(x\right)}=A_{2}+\frac{A_{1}-A_{2}}{1+e^{\left[(X-X_{o})/dX\right]}}$$ (1)

where A_2_ is the final value, A_1_ is the initial value; X_o_ is the center, and *dX* is the time constant.

All boxplots show mean (square), median (bisecting line), bounds of box (75th to 25th percentiles), and outlier range with 1.5 coefficient (whiskers), unless specified. Statistical analyses were performed using GraphPad InStat software (version 3.10; GraphPad Software Inc.) and OriginPro. We used the Kolmogorov-Smirnov method to determine data distribution and Bartlett’s test to determine differences between SDs. Individual tests are described in each of the figure legends.

## Supplementary Materials

### The PDF file includes:

Molecular dynamics supplementary methods Figs. S1 to S9 Tables S1 and S2 Legend for movie S1 References
