# Supplementary material for: Functional and structural basis of a hypermorphic TRPC3 variant
Source: Sci Adv. 2026 Mar 25;12(13):eaec9284. doi: 10.1126/sciadv.aec9284 (PMC13015894; doi:10.1126/sciadv.aec9284)
Supplement: Supplementary file 1 — Molecular dynamics supplementary methods Figs. S1 to S9 Tables S1 and S2 Legend for movie S1 References [file sciadv.aec9284_sm.pdf]

Science Advances
AAAS

Supplementary Materials for

Functional and structural basis of a hypermorphic TRPC3 variant

Briar Bell *et al.*

Corresponding author: Julio F. Cordero-Morales, julio.cordero@uth.tmc.edu

Sci. Adv. **12**, eaec9284 (2026)
DOI: 10.1126/sciadv.aec9284

The PDF file includes:

Molecular dynamics supplementary methods
Figs. S1 to S9
Tables S1 and S2
Legend for movie S1
References

Other Supplementary Material for this manuscript includes the following:

Movie S1

Supplementary Text

Supplementary methods. Molecular dynamics simulations

System setup

A representative snapshot of the overall molecular dynamics (MD) unit cell is shown in **figure 1A of these supplementary methods**. After inserting the coordinates of the missing regions predicted from Alpha Fold 3 (AF3) (*78*) into the cryo-EM structure, and capping the N- and C-termini of each submit, we embed the cryo-EM tetramers into separate 17.1×17.1 nm patches of preequilibrated POPC bilayers (*79*). Embedding is done by aligning the hydrophobic surfaces of the proteins with the lipid hydrophobic core and removing POPC molecules that significantly overlap protein atoms. Each system contains 542 lipids. Membrane-embedded tetramer conformations are then placed in a 17.1×17.1×18 nm cubic boxes and solvated. Waters contained within the space occupied by the POPC bilayer are removed. An ionic strength of 150 mM is set by replacing waters with 326 K^+ and 278 Cl^- ions. The difference in the numbers of positive and negative ions serve to counter the negative charge of -48 eu on each protein. The Δ28 system contains 119,695 waters and T573A/Δ28 contains 119,592 waters. Each system is subjected to energy minimization followed by at least 50 ns of MD with harmonic position restrains (1000 kJ/mol/nm^2) placed on protein heavy atoms. After box dimensions settle in 150 ns, the harmonic restraints are removed by lowering the force constants in four steps (500, 250, 125 and 62 kJ/mol/nm^2) and running each step for 2 ns. The final snapshot is used for starting potential of mean force (PMF) calculations.

Potential of Mean Force (PMF)

To determine the PMFs of Na^+ ions along with pore axes of cryo-EM structures, we use umbrella sampling (*75*) and the weighted histogram analysis method (WHAM) (*76, 80*). The starting conformations for umbrella sampling are obtained from steered molecular dynamics (SMD) (*77*). SMD is initiated from the final snapshot of molecular dynamics (MD). In this snapshot, we replace a water molecule located along the pore axis and in the bulk water region with a Na^+ ion. To maintain charge neutrality, we replace an additional water molecule with Cl^- ion. After adding the two ions, the systems are energy minimized and subjected to 500 ps of MD with harmonic position restraints of 800 kJ/mol/nm^2 placed on Na^+ ions. SMD is then initiated in which the Na^+ ion is pulled along the z-direction towards the gate (center of mass of I670) using a force constant of 800 kJ/mol/nm^2 and a pull rate of 0.001 nm/ps. During SMD, protein backbone atoms are harmonically restrained using a force constant of 30 kJ/mol/nm^2. The movement of the Na^+ ion is also restricted in the XY plane using the XY distance of the four I670 C_α atoms.

The trajectory obtained from SMD is used for extracting starting conformations for umbrella sampling. Umbrella centers are selected at intervals of roughly 1.5 Å, and Na^+ is harmonically restrained at these centers using a force constant of 800 kJ/mol/nm^2 applied only to the z-coordinate. For each system, we use 60 windows, and each window is simulated for 20 ns. The final 10 ns is used for analysis, and statistical uncertainties are estimated using block averaging. For both systems, we initially did not find sufficient overlap (>20%) between some adjacent windows at/near the gate, but this was fixed by using larger force constants of 1600 and 2400 kJ/mol/nm^2 for some windows in which z-coordinate distributions had moved away from their initial locations (6 for Δ28, and 4 for T573A/Δ28). The z-distributions of the windows are illustrated in **supplementary methods figure 1B and C**.

MD parameters

Energy minimization, MD, SMD and umbrella sampling simulations are carried out using GROMACS version 2020.4 (*81*). These simulations are carried out at constant pressure and temperature. Pressure is regulated at 1.01 bar using semi-isotropic coupling, and with a time constant of 1 ps and a compressibility of 4.5 x 10^{-5}. During MD equilibration, we employ Berendsen barostat (*82*), and for SMD and umbrella sampling, we employ the Parinello-Rahman barostat (*83*). Temperature is regulated at 310 K using the velocity rescale thermostat with a coupling time constant of 0.1 ps (*84*). Bonds connecting hydrogen atoms are constrained using the LINCS algorithm (*85*), allowing the use of an integration time step of 2 fs. Integration is carried out using the leap-frog algorithm. Periodic boundary conditions are set in all directions. Electrostatic interactions are computed using particle mesh Ewald (PME) (*86*) with a short-range cutoff of 10 Å, Fourier grid spacing of 1 Å, and a fourth-order interpolation. van der Waals interactions are computed using a cut-off of 10 Å. Neighbor lists are updated using a grid-based scheme.

Water, protein and lipids are described using, respectively, the SPC/E (*87*), amber99sb-ILDN (*88*) and lipid21 force fields (*89*). Titratable amino acids are assigned their default protonation states at pH of 7.4, except for the three cysteines (residues 194, 196, 199) that coordinate the bound Zn^{2+} ion. Parameters of the bound Zn^{2+} ion and the partial charges of the coordinating negatively charged cysteines and H190 are adjusted according to Macchiagodena et al. (*90*) . Na^{+}, K^{+} and Cl^{-} ions are described using Joung and Cheatham parameters (*91*). Development of ion force fields remains an active area of research (*92, 93*), and the Na^{+} parameter set that we chose for computing PMFs does well at modeling the size and structure of its inner coordination shell in the aqueous phase, but its performance in describing interactions with protein remain to be systematically evaluated.

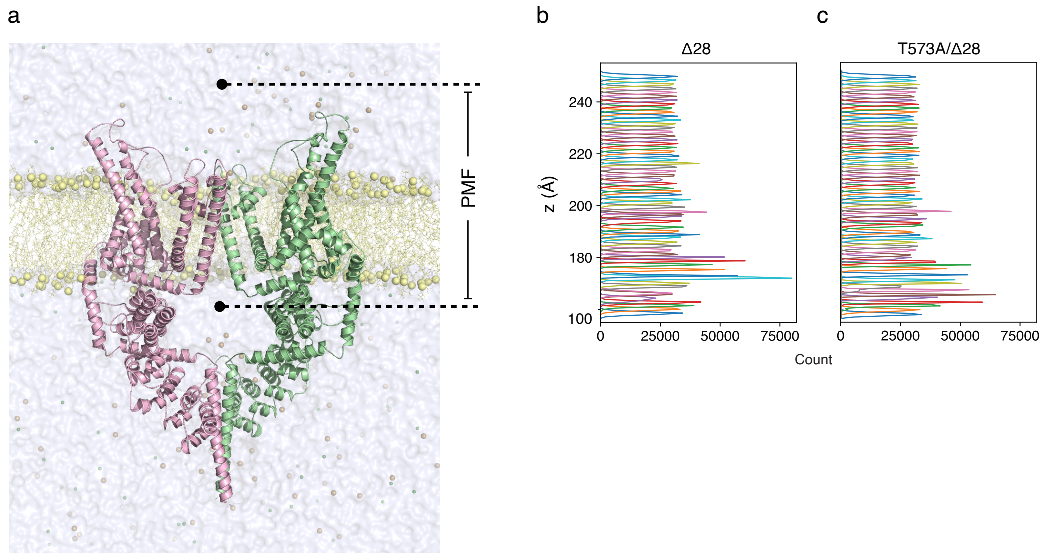

Supplementary methods Fig. 1 (**A**) Lateral view of the MD unit cell. For the sake of clarity, two Δ28 subunits are hidden, lipids/water/ions in front of Δ28 are hidden, and the Δ28 loops are smoothed. Water is shown as a surface, and the brown and green spheres represent K^{+} and Cl^{-} ions,

respectively. The transverse region (z-axis) for which PMF is calculated is also indicated. (**B and C**) Transverse (z) distributions of Na^+ in the different umbrella sampling windows used for PMF calculations. Colors are assigned to only make the different umbrella sampling windows distinguishable from each other. The z-coordinates correspond to the MD reference frame, but those shown in the PMF in the main manuscript have been translated to the cryo-EM reference frame.

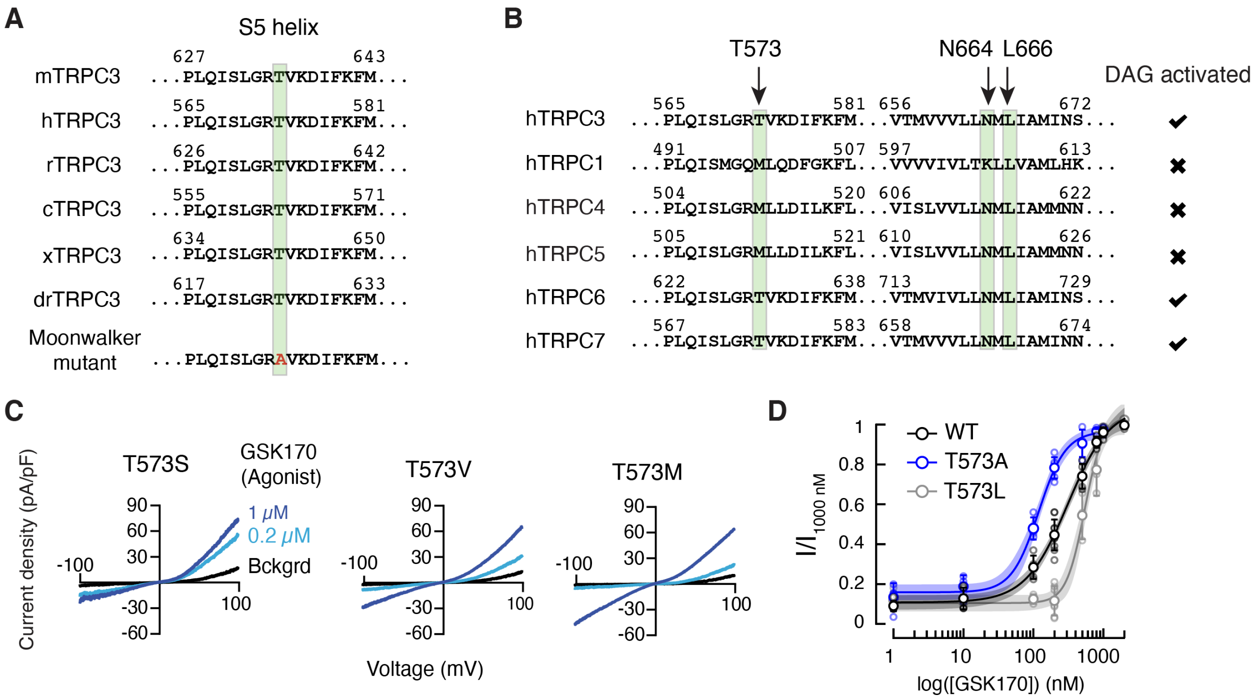

Fig. S1. Data supporting the functional characterization of T573 hTRPC3 mutants. (**A**) S5 helix sequence alignment of TRPC3 channel orthologs (m: *Mus musculus*, h: *Homo sapiens*, r: *Rattus norvegicus*, c: *Gallus gallus domesticus*, x: *Xenopus laevis*, dr: *Danio rerio*). Residue T635 (location of the moonwalker mouse mutation) and T573 in humans is highly conserved across species. (**B**) S6 helix sequence alignment of TRPC subfamily members. Residues T573, N664, and L666 are conserved in TRPC3, TRPC6, and TRPC7. (**C**) Representative current-voltage relationships determined by whole-cell patch-clamp recordings of HEK293 cells expressing T573S, T573V, and T573M hTRPC3 mutants in the absence (Bckgrd) and presence of 0.2 (pale blue) and 1 μM (saturating concentration, blue) GSK1702934A (GSK170, a TRPC3 agonist). (**D**) Normalized current ($I/Imax_{(1000\ nm)}$) GSK170 dose-response profiles of WT hTRPC3, as well as T573A and T573L mutants. A Boltzmann function was fitted to the data. The shadows encompassing the curves indicate the 95% confidence bands for the fit. Enlarged circles are mean ± SD. n = 15 for WT, n = 12 for T573A, and n = 9 for T573L.

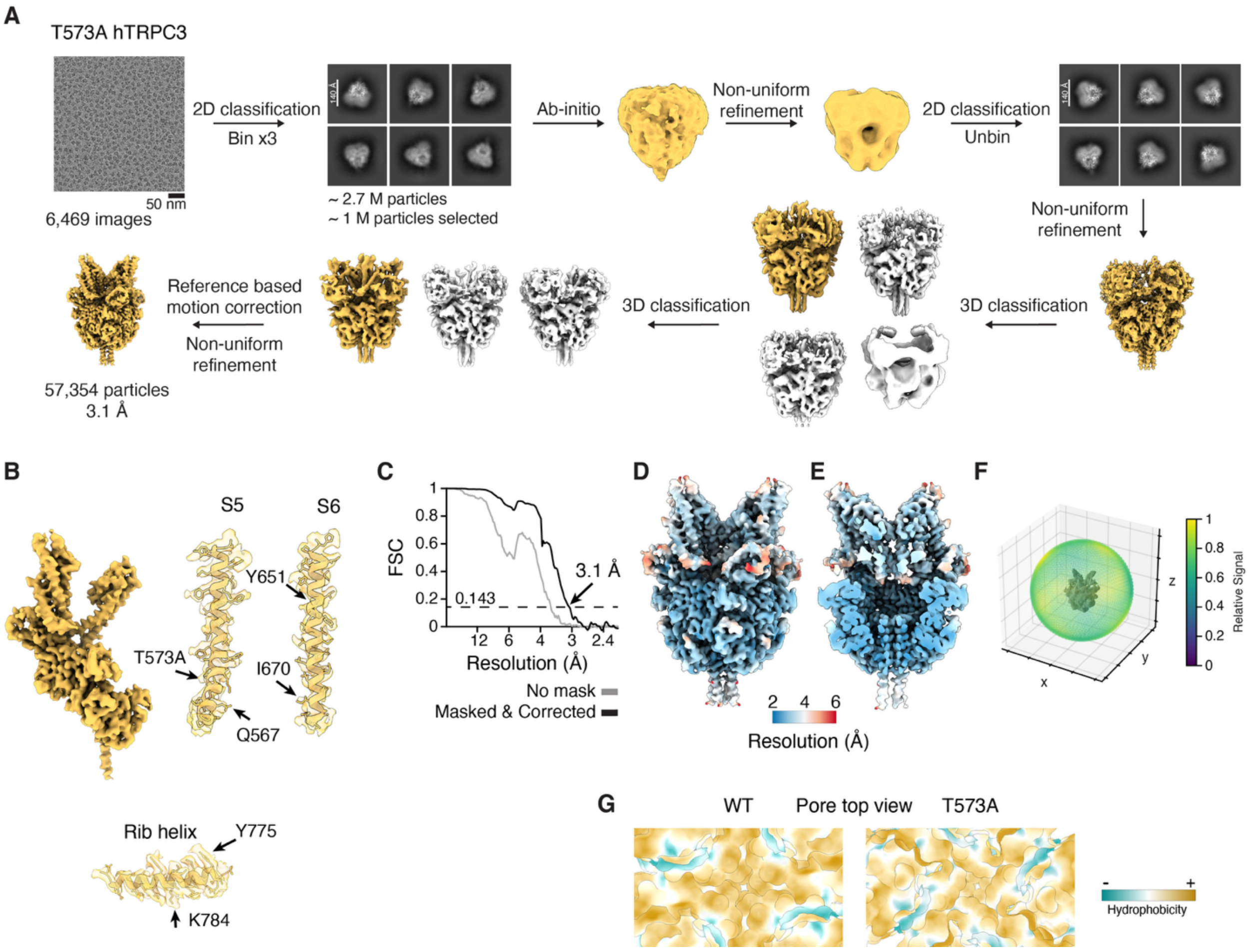

Fig. S2. Data supporting the Cryo-EM data processing, map quality, FSC plot, local resolution, and angular distribution of T573A hTRPC3. (**A**) Cryo-EM data processing. All steps were carried out in CryoSPARC v.4.6 (see methods for details). (**B**) Representative regions for the local fit of the atomic model of T573A hTRPC3 showing S5, S6, and rib helices. (**C**) Corrected Fourier shell correlation (FSC) curve. (**D**) Local resolution. (**E**) Central slice highlighting the local resolution of the pore-forming helices. (**F**) Angular distribution of particles for T573A hTRPC3. PDB 9OLL and EMD-70596. (**G**) Cross-section through the pore showing the lipophilic surface potential of WT and T573A. Surfaces are colored from dark goldenrod (most hydrophobic) to dark cyan (most hydrophilic).

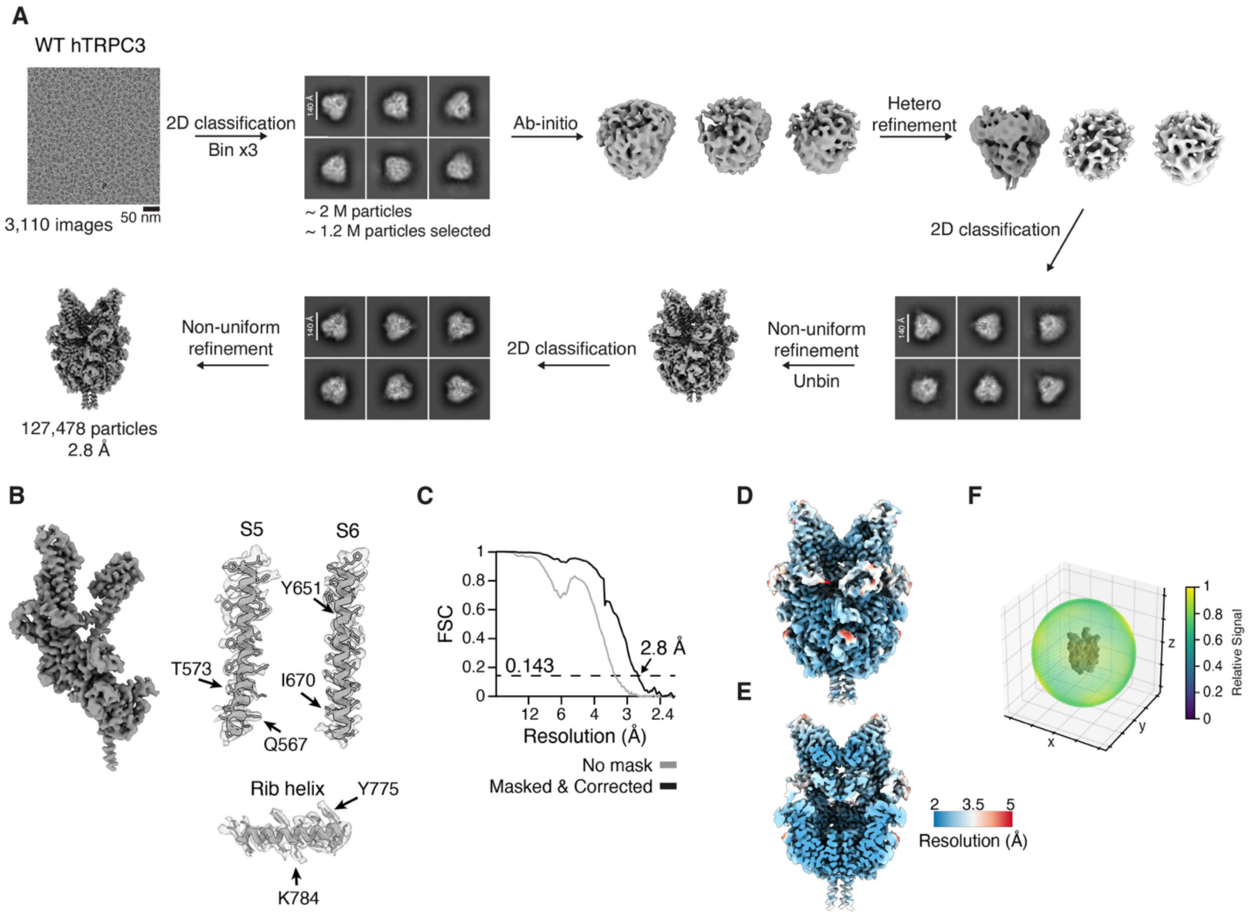

Fig. S3. Data supporting the Cryo-EM data processing, map quality, FSC plot, local resolution, and angular distribution of WT hTRPC3. (**A**) Cryo-EM data processing. All steps were carried out in CryoSPARC v.4.6 (see methods for details). (**B**) Representative regions for the local fit of the atomic model of WT hTRPC3, showing S5, S6, and rib helices. (**C**) Corrected FSC curve. (**D**) Local resolution. (**E**) Central slice highlighting the local resolution of the pore-forming helices. (**F**) Angular distribution of particles for WT hTRPC3. PDB 9OLK and EMD-70595.

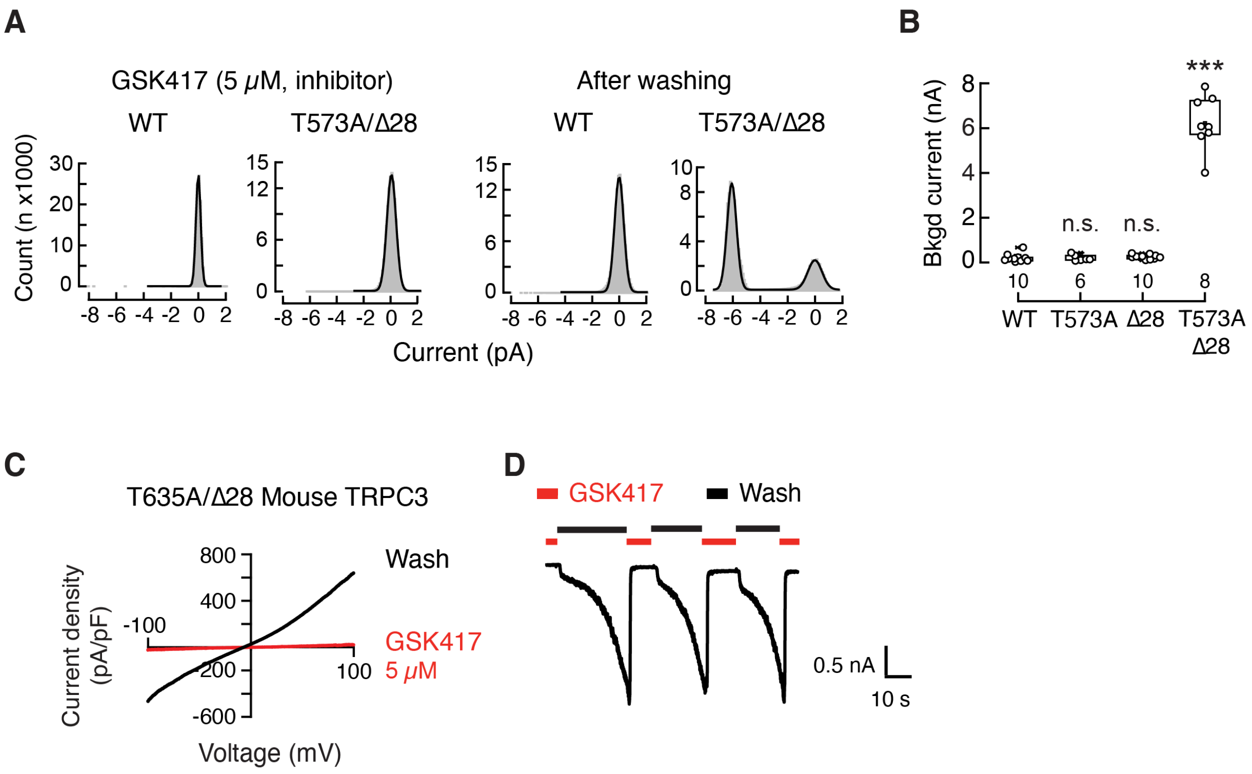

Fig. S4. Data supporting the functional characterization of the T573A/Δ28 hTRPC3. (**A**) All-point amplitude histograms of WT and T573A/Δ28 hTRPC3 single-channel currents in the presence of GSK417 (5 μM) and after washout. Histograms were generated from recordings shown in **Fig. 2J**. (**B**) Boxplots of background WT, T573A, Δ28, and T573A/Δ28 hTRPC3 currents obtained by whole-cell patch-clamp recordings (+100 mV). One-way ANOVA ($F = 206.71$, $p = 0$) and Bonferroni multiple-comparisons test. (**C**) Representative current-voltage relationships of HEK293 cells expressing T635A/Δ28 mouse TRPC3 in the presence of GSK417 (red) and after washout (black). (**D**) Representative time course of whole-cell patch-clamp recordings at -60 mV of the T635A/Δ28 mouse TRPC3 construct in the presence of GSK417 (red) and after washout (black). ∗∗∗$p < 0.001$. n.s. indicates not significant.

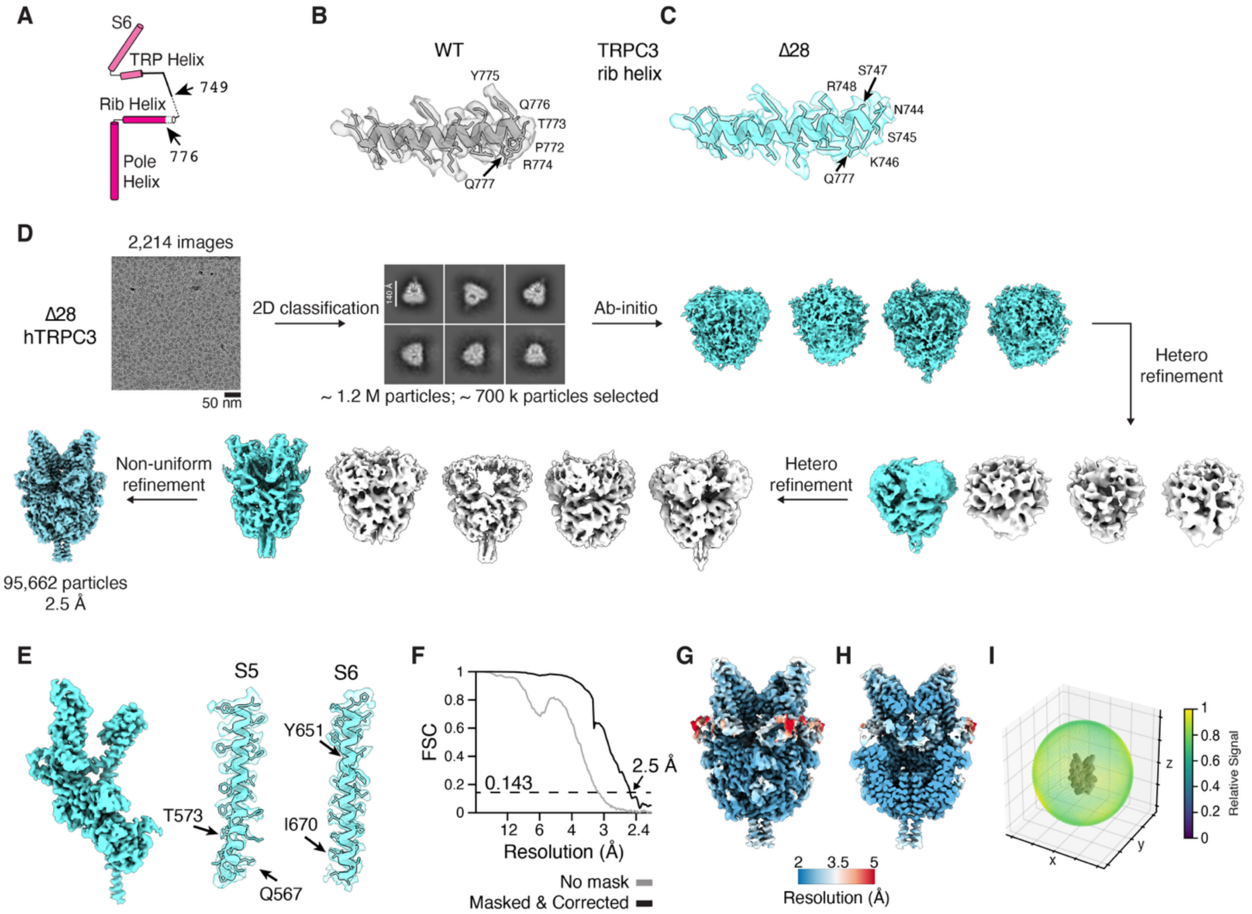

Fig. S5. Data supporting the Cryo-EM data processing, map quality, FSC plot, local resolution, and angular distribution of Δ28 hTRPC3. (**A**) One subunit cartoon depicting the changes in the C-terminal loop and rib helix of hTRPC3 to generate Δ28. (**B and C**) Cryo-EM densities of WT and Δ28 hTRPC3 rib helices, with the protein shown in ribbon, depicting residues missing in the Δ28 constructs (e.g., Y775). (**D**) Cryo-EM data processing. All steps were carried out in CryoSPARC v.4.6 (see methods for details). (**E**) Representative regions for the local fit of the atomic model of Δ28 hTRPC3, showing S5 and S6 helices. (**F**) Corrected FSC curve. (**G**) Local resolution. (**H**) Central slice highlighting the local resolution of the pore-forming helices. (**I**) Angular distribution of particles for Δ28 hTRPC3. PDB 9OLM and EMD-70597.

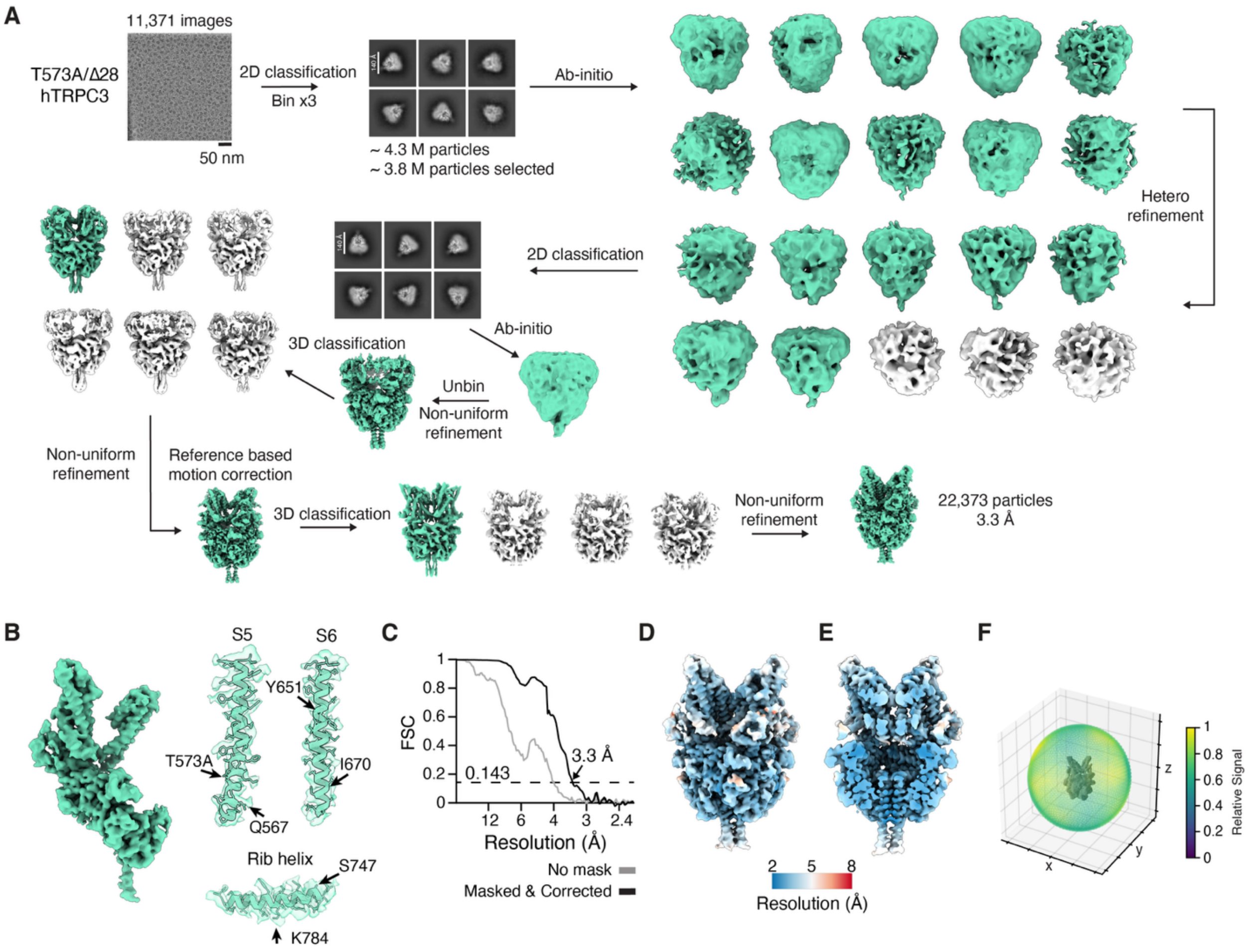

Fig. S6. Data supporting the Cryo-EM data processing, map quality, FSC plot, local resolution, and angular distribution of T573A/Δ28 hTRPC3. (**A**) Cryo-EM data processing. All steps were carried out in CryoSPARC v.4.6 (see methods for details). (**B**) Representative regions for the local fit of the atomic model of T573A/Δ28 hTRPC3, showing S5, S6, and rib helices. (**C**) Corrected FSC curve. (**D**) Local resolution. (**E**) Central slice highlighting the local resolution of the pore-forming helices. (**F**) Angular distribution of particles for T573A/Δ28 hTRPC3. PDB 9OPU and EMD-70724.

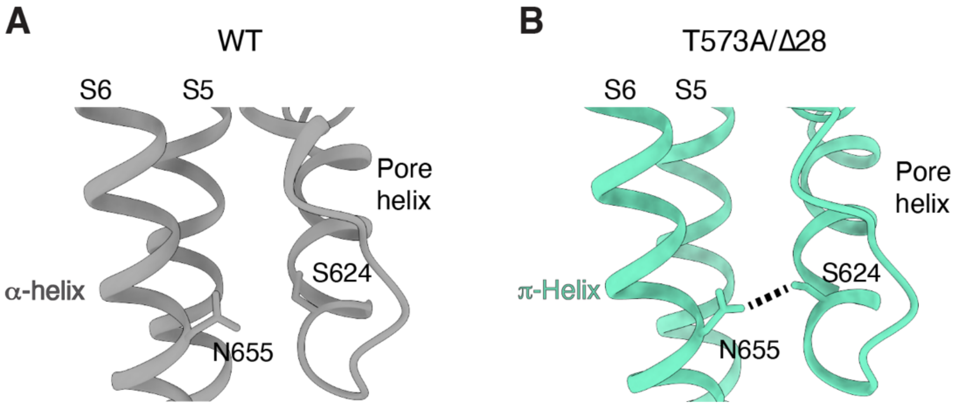

Fig. S7. Data supporting the structural characterization of the T573A/Δ28 mutant. (**A and B**) Expanded side view of the WT and T573A/Δ28 S5, S6, and pore helix regions, illustrating a hydrogen bond interaction stabilizing the π helix within the T573A/Δ28 S6 helix.

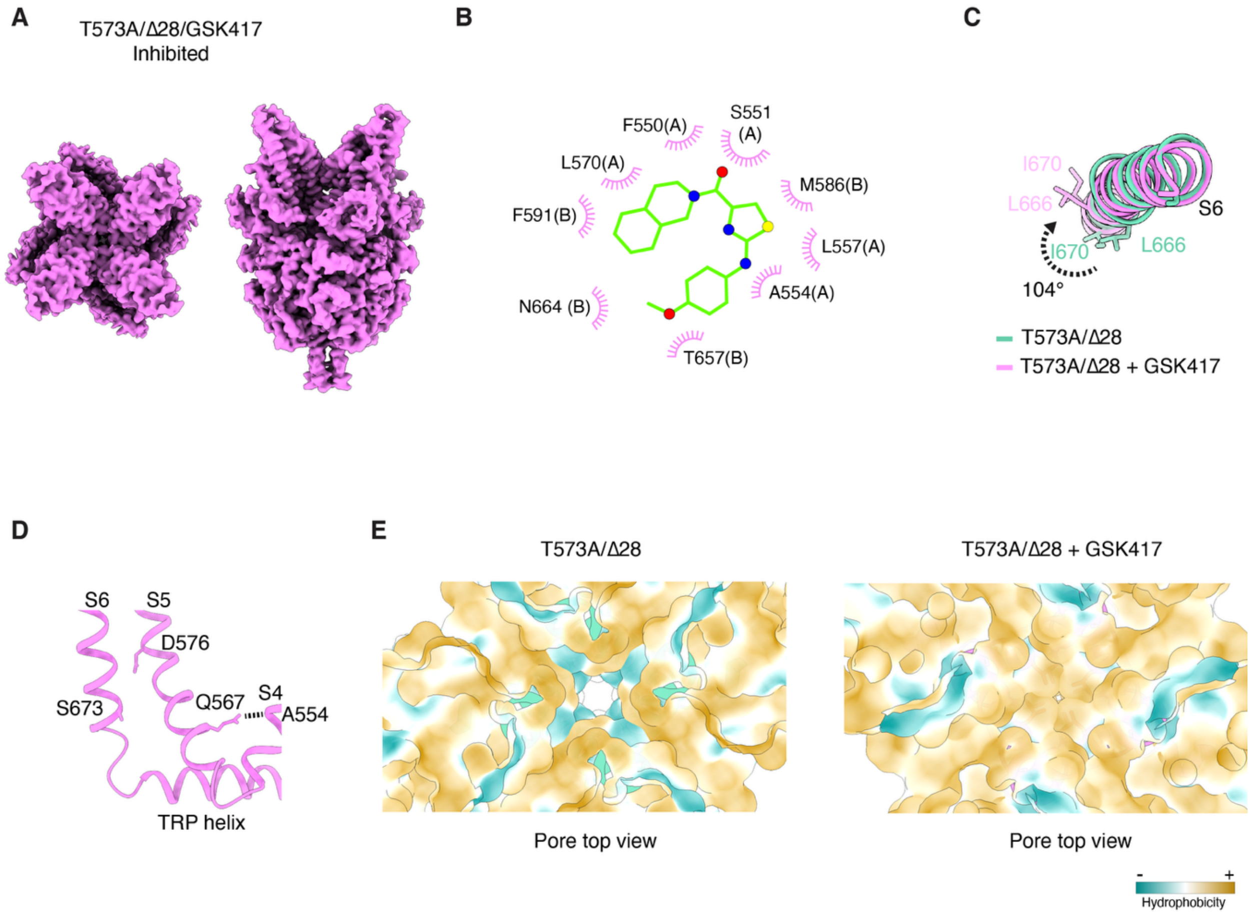

Fig. S8. Data supporting the structural characterization of the T573A/Δ28 hTRPC3 in complex with GSK417. (**A**) Top and side view of the cryo-EM map of T573A/Δ28/GSK417 hTRPC3, PDB 9OLX and EMD-70601. (**B**) LigPlot schematic of GSK417 with the T573A/Δ28 mutant, amino acid residues forming the GSK417 binding pocket are highlighted. GSK417 backbone carbon (green), oxygen (red), nitrogen (blue), and sulfur atoms (yellow) are shown. (**C**) Top view superposition of the S6 helices depicting the rotation of hydrophobic residues back toward the permeation pathway. (**D**) Expanded side view of the bundle crossing shows the hydrogen bond interaction between the S4 and S5 helices in the T573A/Δ28/GSK417 structure. (**E**) Cross-section through the pore showing the lipophilic surface potential of T573A/Δ28 and T573A/Δ28/GSK417. Surfaces are colored from dark goldenrod (most hydrophobic) to dark cyan (most hydrophilic).

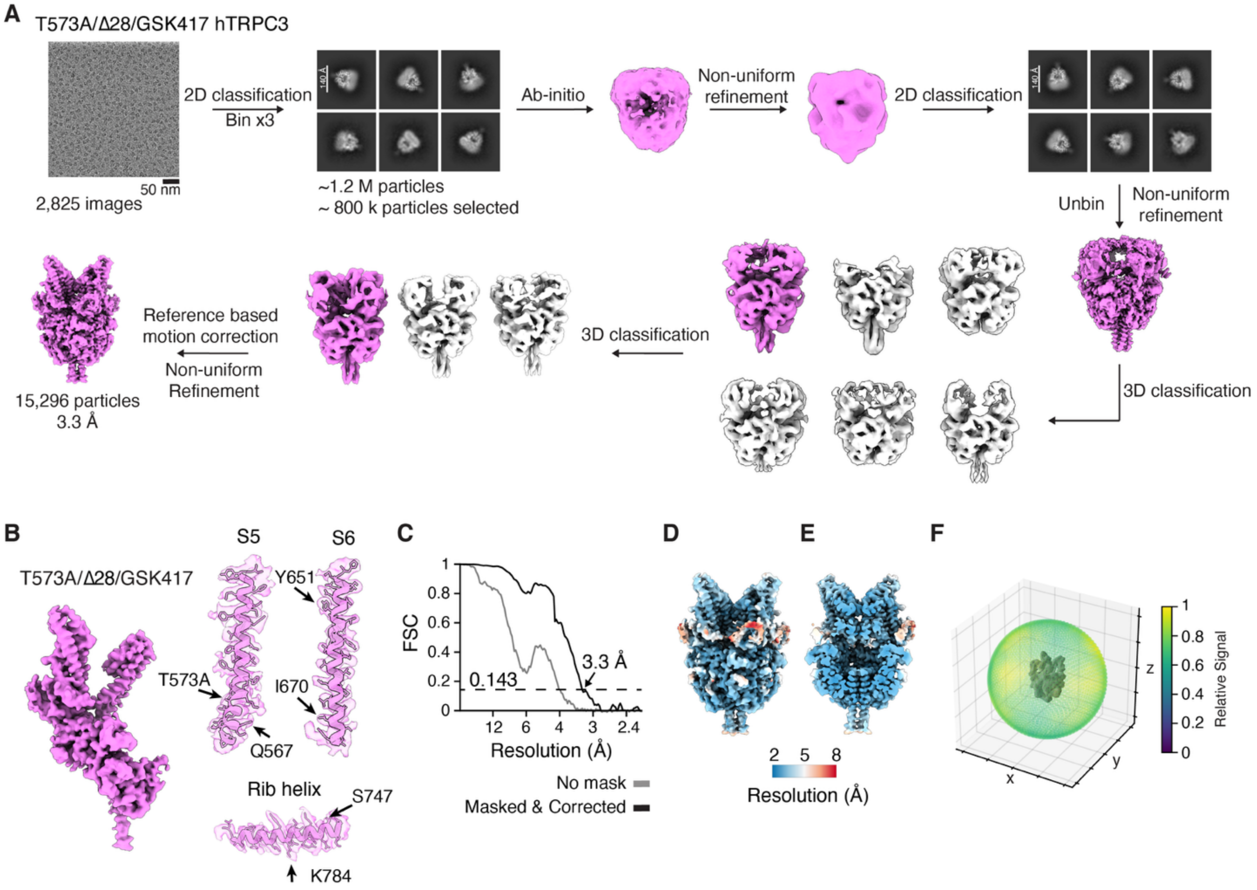

Fig. S9. Data supporting the Cryo-EM data processing, map quality, FSC plot, local resolution, and angular distribution of T573A/Δ28/GSK417 hTRPC3. (**A**) Cryo-EM data processing. All steps were performed in CryoSPARC v.4.6 (see Methods for details). (**B**) Representative regions for the local fit of the atomic model of T573A/Δ28/GSK417 hTRPC3, showing S5, S6, and rib helices. (**C**) Corrected FSC curve. (**D**) Local resolution. (**E**) Central slice highlighting the local resolution of the pore-forming helices. (**F**) Angular distribution of particles for T573A/Δ28/GSK417 hTRPC3. PDB 9OLX and EMD-70601.

Table S1. Cryo-EM data collection, refinement, and validation statistics.

	WT TRPC3 (EMD-70595) (PDB 9OLK)	Δ28 (EMD-70597) (PDB 9OLM)	T573A (EMD-70596) (PDB 9OLL)	T573A/Δ28 (EMDB-70724) (PDB 9OPU)	T573A/Δ28/ GSK417 (EMD-70601) (PDB 9OLX)
**<u>Data collection and processing</u>**					
Microscope			Krios G3		
Detector			K2		
Magnification			130K		
Voltage (kV)			300		
Electron exposure (e–/Å^2)			50		
Defocus range (μm)			0.8 - 2.2		
Pixel size (Å)			1.08		
Symmetry imposed			C4		
Number of movies collected	3,110	2,214	6,469	11,371	2,825
Initial particle images (no.)	~2 M	~ 1.2 M	~2.7 M	~4.3 M	~1.2 M
Final particle images (no.)	127,478	95,662	57,354	22,373	15,296
Map resolution (Å)	2.8	2.5	3.1	3.3	3.3
FSC threshold					
Map sharpening *B* factor (Å^2)	82.7	65.6	86.4	79.4	75.8
**<u>Model composition</u>**					
Non-hydrogen atoms	24,568	24,528	24,564	24,480	24,592
Protein residues	2,980	2,980	2,980	2,980	2,980
Ligands	12	12	12	12	16
**<u>Refinement</u>**					
Initial model used (PDB code)	7DXD	9OLK	9OLK	9OLM	9OLM
B factors (Å^2)					
Protein	78.66	39.10	65.91	86.28	82.35
Ligand	18.93	75.61	36.61	115.22	26.13
R.m.s. deviations					
Bond lengths (Å)	0.011	0.007	0.010	0.011	0.157
Bond angles (°)	1.621	1.007	1.455	1.610	2.488
Validations					
MolProbity score	1.68	1.73	2.03	2.21	1.96
Clashscore	4.72	5.65	7.79	6.46	8.76
Poor rotamers (%)	1.07	1.07	1.67	1.98	1.07
Ramachandran plot					
Favored (%)	93.64	94.05	93.40	86.84	92.56
Allowed (%)	5.82	5.01	6.19	11.40	6.09
Disallowed	0.54	0.95	0.41	1.76	1.35
Model vs. Data					
CC (mask)	0.75	0.80	0.70	0.76	0.78
CC (box)	0.61	0.68	0.63	0.68	0.70
CC (peaks)	0.57	0.66	0.55	0.60	0.63
CC (volume)	0.73	0.78	0.67	0.74	0.75
Mean CC for ligands	0.59	0.55	0.42	0.46	0.58

Table S2. Model-to-model RMSD comparison.

	WT TRPC3 (EMD-70595) (PDB 9OLK)	Δ28 (EMD-70597) (PDB 9OLM)	T573A (EMD-70596) (PDB 9OLL)	T573A/Δ28 (EMDB-70724) (PDB 9OPU)	T573A/Δ28/ GSK417 (EMD-70601) (PDB 9OLX)	WT TRPC3 (PDB 6CUD)	WT TRPC3 (PDB 5ZBG)
WT TRPC3		0.975	1.040	1.784	1.972	2.952	1.486
Δ28	0.975		0.976	1.522	1.590		
T573A	1.040	0.976		1.637	1.637		
T573A/Δ28	1.784	1.522	1.637		1.975		
T573A/Δ28/GSK417	1.972	1.590	1.637	1.975			

Movie S1. Structural changes between closed, open, and inhibited human WT TRPC3 and a disease-causing mutation A morph between closed (WT), open (T573A/Δ28), and inhibited (T573A/Δ28/GSK417) states of human TRPC3. The TRPC3 pore domain is depicted from the extracellular side, highlighting the expansion of the permeation pathway, and a parallel view, showing the α-to-π helical transition in the S6 during channel opening, along with the rotation of the hydrophobic residues L666 and I670. Also, a similar representation highlights the conformational changes during channel inhibition. Residues are shown in stick representation.

REFERENCES

1. S. Jayadev, T. D. Bird, Hereditary ataxias: Overview. *Genet. Med.* **15**, 673–683 (2013).

2. A. H. Koeppen, The pathogenesis of spinocerebellar ataxia. *Cerebellum* **4**, 62–73 (2005).

3. T. A. Zesiewicz, G. Wilmot, S. H. Kuo, S. Perlman, P. E. Greenstein, S. H. Ying, T. Ashizawa, S. H. Subramony, J. D. Schmahmann, K. P. Figueroa, H. Mizusawa, L. Schols, J. D. Shaw, R. M. Dubinsky, M. J. Armstrong, G. S. Gronseth, K. L. Sullivan, Comprehensive systematic review summary: Treatment of cerebellar motor dysfunction and ataxia [RETIRED]: Report of the Guideline Development, Dissemination, and Implementation Subcommittee of the American Academy of Neurology. *Neurology* **90**, 464–471 (2018).

4. D. D. Bushart, V. G. Shakkottai, Ion channel dysfunction in cerebellar ataxia. *Neurosci. Lett.* **688**, 41–48 (2019).

5. L. Schöls, P. Bauer, T. Schmidt, T. Schulte, O. Riess, Autosomal dominant cerebellar ataxias: Clinical features, genetics, and pathogenesis. *Lancet Neurol.* **3**, 291–304 (2004).

6. M. Anheim, C. Tranchant, M. Koenig, The autosomal recessive cerebellar ataxias. *N. Engl. J. Med.* **366**, 636–646 (2012).

7. E. B. Becker, P. L. Oliver, M. D. Glitsch, G. T. Banks, F. Achilli, A. Hardy, P. M. Nolan, E. M. Fisher, K. E. Davies, A point mutation in TRPC3 causes abnormal Purkinje cell development and cerebellar ataxia in moonwalker mice. *Proc. Natl. Acad. Sci. U.S.A.* **106**, 6706–6711 (2009).

8. G. Sekerková, J. A. Kim, M. J. Nigro, E. B. Becker, J. Hartmann, L. Birnbaumer, E. Mugnaini, M. Martina, Early onset of ataxia in moonwalker mice is accompanied by complete ablation of type II unipolar brush cells and Purkinje cell dysfunction. *J. Neurosci.* **33**, 19689–19694 (2013).

9. G. Sekerkova, S. Kilic, Y.-H. Cheng, N. Fredrick, A. Osmani, H. Kim, P. Opal, M. Martina, Phenotypical, genotypical and pathological characterization of the moonwalker mouse, a model of ataxia. *Neurobiol. Dis.* **195**, 106492 (2024).

10. J. P. Jeon, C. Hong, E. J. Park, J. H. Jeon, N. H. Cho, I. G. Kim, H. Choe, S. Muallem, H. J. Kim, I. So, Selective Gαi subunits as novel direct activators of transient receptor potential canonical $(TRPC)_4$ and $TRPC_5$ channels. *J. Biol. Chem.* **287**, 17029–17039 (2012).

11. T. Okada, R. Inoue, K. Yamazaki, A. Maeda, T. Kurosaki, T. Yamakuni, I. Tanaka, S. Shimizu, K. Ikenaka, K. Imoto, Y. Mori, Molecular and functional characterization of a novel mouse transient receptor potential protein homologue TRP7. Ca^{2+}-permeable cation channel that is constitutively activated and enhanced by stimulation of G protein-coupled receptor. *J. Biol. Chem.* **274**, 27359–27370 (1999).

12. T. Hofmann, A. G. Obukhov, M. Schaefer, C. Harteneck, T. Gudermann, G. Schultz, Direct activation of human TRPC6 and TRPC3 channels by diacylglycerol. *Nature* **397**, 259–263 (1999).

13. E. Yildirim, L. Birnbaumer, "TRPC2: Molecular biology and functional importance" in *Transient Receptor Potential (TRP) Channels*, V. Flockerzi, B. Nilius, Eds. (Springer Berlin Heidelberg, 2007), pp. 53–75.

14. A. Riccio, A. D. Medhurst, C. Mattei, R. E. Kelsell, A. R. Calver, A. D. Randall, C. D. Benham, M. N. Pangalos, mRNA distribution analysis of human TRPC family in CNS and peripheral tissues. *Brain Res. Mol. Brain Res.* **109**, 95–104 (2002).

15. C. M. Diebolt, D. Schaudien, K. Junker, G. Krasteva-Christ, T. Tschernig, C. N. Englisch, New insights in the renal distribution profile of TRPC3—Of mice and men. *Ann. Anat.* **252**, 152192 (2024).

16. P. Eder, D. Probst, C. Rosker, M. Poteser, H. Wolinski, S. D. Kohlwein, C. Romanin, K. Groschner, Phospholipase C-dependent control of cardiac calcium homeostasis involves a TRPC3-NCX1 signaling complex. *Cardiovasc. Res.* **73**, 111–119 (2007).

17. D. E. Clapham, TRP channels as cellular sensors. *Nature* **426**, 517–524 (2003).

18. J. Hartmann, A. Konnerth, TRPC3-dependent synaptic transmission in central mammalian neurons. *J. Mol. Med.* **93**, 983–989 (2015).

19. J. Hartmann, E. Dragicevic, H. Adelsberger, H. A. Henning, M. Sumser, J. Abramowitz, R. Blum, A. Dietrich, M. Freichel, V. Flockerzi, L. Birnbaumer, A. Konnerth, TRPC3 channels are required for synaptic transmission and motor coordination. *Neuron* **59**, 392–398 (2008).

20. Y. Kim, A. C. Wong, J. M. Power, S. F. Tadros, M. Klugmann, A. J. Moorhouse, P. P. Bertrand, G. D. Housley, Alternative splicing of the TRPC3 ion channel calmodulin/IP3 receptor-binding domain in the hindbrain enhances cation flux. *J. Neurosci.* **32**, 11414–11423 (2012).

21. J. M. E. Cederholm, Y. Kim, G. von Jonquieres, G. D. Housley, Human brain region-specific alternative splicing of TRPC3, the type 3 canonical transient receptor potential non-selective cation channel. *Cerebellum* **18**, 536–543 (2019).

22. F. Sierra-Valdez, C. M. Azumaya, L. O. Romero, T. Nakagawa, J. F. Cordero-Morales, Structure-function analyses of the ion channel TRPC3 reveal that its cytoplasmic domain allosterically modulates channel gating. *J. Biol. Chem.* **293**, 16102–16114 (2018).

23. J. Tang, Y. Lin, Z. Zhang, S. Tikunova, L. Birnbaumer, M. X. Zhu, Identification of common binding sites for calmodulin and inositol 1,4,5-trisphosphate receptors on the carboxyl termini of Trp channels. *J. Biol. Chem.* **276**, 21303–21310 (2001).

24. Z. Zhang, J. Tang, S. Tikunova, J. D. Johnson, Z. Chen, N. Qin, A. Dietrich, E. Stefani, L. Birnbaumer, M. X. Zhu, Activation of Trp3 by inositol 1,4,5-trisphosphate receptors through displacement of inhibitory calmodulin from a common binding domain. *Proc. Natl. Acad. Sci. U.S.A.* **98**, 3168–3173 (2001).

25. D. Vinayagam, D. Quentin, J. Yu-Strzelczyk, O. Sitsel, F. Merino, M. Stabrin, O. Hofnagel, M. Yu, M. W. Ledeboer, G. Nagel, G. Malojcic, S. Raunser, Structural basis of TRPC4 regulation by calmodulin and pharmacological agents. *eLife* **9**, e60603 (2020).

26. V. K. Boda, N. Yasmen, J. Jiang, W. Li, Pathophysiological significance and modulation of the transient receptor potential canonical 3 ion channel. *Med. Res. Rev.* **44**, 2510–2544 (2024).

27. Q. Tang, W. Guo, L. Zheng, J. X. Wu, M. Liu, X. Zhou, X. Zhang, L. Chen, Structure of the receptor-activated human TRPC6 and TRPC3 ion channels. *Cell Res.* **28**, 746–755 (2018).

28. Y. Chen, J. Zang, W. Guo, J. Xu, M. Wei, L. Quan, M. Zhu, X. Zhao, H. Peng, Y. Wan, L. Chen, Structural mechanism of the agonist binding on human TRPC3 channel. *Nat. Commun.* **16**, 9343 (2025).

29. M. Hagimori, T. Murakami, K. Shimizu, M. Nishida, T. Ohshima, T. Mukai, Synthesis of radioiodinated probes to evaluate the biodistribution of a potent TRPC3 inhibitor. *MedChemComm* **7**, 1003–1006 (2016).

30. H. Wang, X. Cheng, J. Tian, Y. Xiao, T. Tian, F. Xu, X. Hong, M. X. Zhu, TRPC channels: Structure, function, regulation and recent advances in small molecular probes. *Pharmacol. Ther.* **209**, 107497 (2020).

31. A. Dulneva, S. Lee, P. L. Oliver, K. Di Gleria, B. M. Kessler, K. E. Davies, E. B. Becker, The mutant *Moonwalker* TRPC3 channel links calcium signaling to lipid metabolism in the developing cerebellum. *Hum. Mol. Genet.* **24**, 4114–4125 (2015).

32. M. F. Ibrahim, E. B. E. Becker, “Moonwalker mouse” in *Essentials of Cerebellum and Cerebellar Disorders: A Primer For Graduate Students*, D. L. Gruol, N. Koibuchi, M. Manto, M. Molinari, J. D. Schmahmann, Y. Shen, Eds. (Springer International Publishing, 2023), pp. 441–447.

33. M. Munakata, H. Shirakawa, K. Nagayasu, J. Miyanohara, T. Miyake, T. Nakagawa, H. Katsuki, S. Kaneko, Transient receptor potential canonical 3 inhibitor Pyr3 improves outcomes and attenuates astrogliosis after intracerebral hemorrhage in mice. *Stroke* **44**, 1981–1987 (2013).

34. S. Kiyonaka, K. Kato, M. Nishida, K. Mio, T. Numaga, Y. Sawaguchi, T. Yoshida, M. Wakamori, E. Mori, T. Numata, M. Ishii, H. Takemoto, A. Ojida, K. Watanabe, A. Uemura, H. Kurose, T. Morii, T. Kobayashi, Y. Sato, C. Sato, I. Hamachi, Y. Mori, Selective and direct inhibition of TRPC3 channels underlies biological activities of a pyrazole compound. *Proc. Natl. Acad. Sci. U.S.A.* **106**, 5400–5405 (2009).

35. H.-K. Ting, Y.-C. Dou, Y.-H. Lin, T.-M. Chen, Y.-L. Tsai, W.-C. Tsai, S.-T. Wu, Y. Chen, Pyr3 inhibits cell viability and PKCα activity to suppress migration in human bladder cancer cells. *Eur. J. Pharmacol.* **988**, 177235 (2025).

36. P. Sherkhane, J. P. Kapfhammer, The plasma membrane Ca^{2+}-ATPase2 (PMCA2) is involved in the regulation of Purkinje cell dendritic growth in cerebellar organotypic slice cultures. *Neural Plast.* **2013**, 321685 (2013).

37. T. P. Stauffer, D. Guerini, E. Carafoli, Tissue distribution of the four gene products of the plasma membrane Ca^{2+} pump. A study using specific antibodies. *J. Biol. Chem.* **270**, 12184–12190 (1995).

38. S. Radmard, T. A. Zesiewicz, S. H. Kuo, Evaluation of cerebellar ataxic patients. *Neurol. Clin.* **41**, 21–44 (2023).

39. D. M. Wilson III, M. R. Cookson, L. Van Den Bosch, H. Zetterberg, D. M. Holtzman, I. Dewachter, Hallmarks of neurodegenerative diseases. *Cell* **186**, 693–714 (2023).

40. B. Wolozin, C. Gabel, A. Ferree, M. Guillily, A. Ebata, Watching worms whither: Modeling neurodegeneration in *C. elegans*. *Prog. Mol. Biol. Transl. Sci.* **100**, 499–514 (2011).

41. K. A. Caldwell, C. W. Willicott, G. A. Caldwell, Modeling neurodegeneration in *Caenorhabditis elegans*. *Dis. Model. Mech.* **13**, dmm046110 (2020).

42. A. Yugeta, H. Arai, D. Takahashi, N. Haruta, A. Sugimoto, H. Arimoto, *C. elegans* ATG-5 mutants associated with ataxia. *MicroPubl. Biol.* **2023**, doi: 10.17912/micropub.biology.000792 (2023).

43. M. Chalfie, E. Wolinsky, The identification and suppression of inherited neurodegeneration in *Caenorhabditis elegans*. *Nature* **345**, 410–416 (1990).

44. J. M. Kaplan, H. R. Horvitz, A dual mechanosensory and chemosensory neuron in Caenorhabditis elegans. *Proc. Natl. Acad. Sci. U.S.A.* **90**, 2227–2231 (1993).

45. C. I. Bargmann, Chemosensation in C. elegans. *WormBook* , 1–29 (2006).

46. C. Fan, W. Choi, W. Sun, J. Du, W. Lu, Structure of the human lipid-gated cation channel TRPC3. *eLife* **7**, e36852 (2018).

47. W. Guo, Q. Tang, M. Wei, Y. Kang, J.-X. Wu, L. Chen, Structural mechanism of human TRPC3 and TRPC6 channel regulation by their intracellular calcium-binding sites. *Neuron* **110**, 1023–1035.e5 (2022).

48. J. Hu, S. J. Park, T. Walter, I. J. Orozco, G. O'Dea, X. Ye, J. Du, W. Lu, Physiological temperature drives TRPM4 ligand recognition and gating. *Nature* **630**, 509–515 (2024).

49. J. Zhao, J. V. Lin King, C. E. Paulsen, Y. Cheng, D. Julius, Irritant-evoked activation and calcium modulation of the TRPA1 receptor. *Nature* **585**, 141–145 (2020).

50. K. Zhang, D. Julius, Y. Cheng, Structural snapshots of TRPV1 reveal mechanism of polymodal functionality. *Cell* **184**, 5138–5150.e12 (2021).

51. Y. Yin, F. Zhang, S. Feng, K. J. Butay, M. J. Borgnia, W. Im, S. Y. Lee, Activation mechanism of the mouse cold-sensing TRPM8 channel by cooling agonist and PIP_2. *Science* **378**, eadd1268 (2022).

52. P. Schmiege, M. Fine, G. Blobel, X. Li, Human TRPML1 channel structures in open and closed conformations. *Nature* **550**, 366–370 (2017).

53. L. Zubcevic, S. Y. Lee, The role of π-helices in TRP channel gating. *Curr. Opin. Struct. Biol.* **58**, 314–323 (2019).

54. I. A. Talyzina, K. D. Nadezhdin, A. I. Sobolevsky, Forty sites of TRP channel regulation. *Curr. Opin. Chem. Biol.* **84**, 102550 (2025).

55. M. A. Kasimova, A. T. Yazici, Y. Yudin, D. Granata, M. L. Klein, T. Rohacs, V. Carnevale, A hypothetical molecular mechanism for TRPV1 activation that invokes rotation of an S6 asparagine. *J. Gen. Physiol.* **150**, 1554–1566 (2018).

56. L. Zubcevic, M. A. Herzik Jr., M. Wu, W. F. Borschel, M. Hirschi, A. S. Song, G. C. Lander, S.-Y. Lee, Conformational ensemble of the human TRPV3 ion channel. *Nat. Commun.* **9**, 4773 (2018).

57. Y. Yin, C. G. Park, S. Feng, Z. Guan, H. J. Lee, F. Zhang, K. Sharma, M. J. Borgnia, W. Im, S. Y. Lee, Molecular basis of neurosteroid and anticonvulsant regulation of TRPM3. *Nat. Struct. Mol. Biol.* **32**, 828–840 (2025).

58. Y. Gao, E. Cao, D. Julius, Y. Cheng, TRPV1 structures in nanodiscs reveal mechanisms of ligand and lipid action. *Nature* **534**, 347–351 (2016).

59. E. Vazquez, M. A. Valverde, A review of TRP channels splicing. *Semin. Cell Dev. Biol.* **17**, 607–617 (2006).

60. R. Caires, B. Bell, J. Lee, L. O. Romero, V. Vásquez, J. F. Cordero-Morales, Deficiency of inositol monophosphatase activity decreases phosphoinositide lipids and enhances TRPV1 function in vivo. *J. Neurosci.* **41**, 408–423 (2021).

61. E. O. Gracheva, J. F. Cordero-Morales, J. A. González-Carcacía, N. T. Ingolia, C. Manno, C. I. Aranguren, J. S. Weissman, D. Julius, Ganglion-specific splicing of TRPV1 underlies infrared sensation in vampire bats. *Nature* **476**, 88–91 (2011).

62. E. B. E. Becker, From mice to men: TRPC3 in cerebellar ataxia. *Cerebellum* **16**, 877–879 (2017).

63. S. M. Neuner, L. A. Wilmott, K. A. Hope, B. Hoffmann, J. A. Chong, J. Abramowitz, L. Birnbaumer, K. M. O'Connell, A. K. Tryba, A. S. Greene, C. Savio Chan, C. C. Kaczorowski, TRPC3 channels critically regulate hippocampal excitability and contextual fear memory. *Behav. Brain Res.* **281**, 69–77 (2015).

64. S. Zhang, L. O. Romero, S. Deng, J. Wang, Y. Li, L. Yang, D. J. Hamilton, D. D. Miller, F. F. Liao, J. F. Cordero-Morales, Z. Wu, W. Li, Discovery of a highly selective and potent TRPC3 inhibitor with high metabolic stability and low toxicity. *ACS Med. Chem. Lett.* **12**, 572–578 (2021).

65. J. Wang, S. Zhang, V. K. Boda, H. Chen, H. Park, K. Parmar, D. Ma, D. D. Miller, B. Meibohm, J. Du, F.-F. Liao, Z. Wu, W. Li, Discovery of a potent and selective TRPC3 antagonist with neuroprotective effects. *Bioorg. Med. Chem.* **117**, 118021 (2025).

66. Z. Wang, D. Ding, J. Wang, L. Chen, Q. Dong, M. Khamrai, Y. Zhou, A. Ishii, K. Sakata, W. Li, J. Du, T. Vaithianathan, F. M. Zhou, F.-F. Liao, Soluble β-amyloid oligomers selectively upregulate TRPC3 in excitatory neurons via calcineurin-coupled NFAT. *Cells* **14**, 843 (2025).

67. M. Liao, E. Cao, D. Julius, Y. Cheng, Structure of the TRPV1 ion channel determined by electron cryo-microscopy. *Nature* **504**, 107–112 (2013).

68. S. Brenner, The genetics of *Caenorhabditis elegans*. *Genetics* **77**, 71–94 (1974).

69. V. Vasquez, Using *C. elegans* to study the effects of toxins in sensory ion channels in vivo. *Methods Mol. Biol.* **2068**, 225–238 (2019).

70. C. Frøkjær-Jensen, M. W. Davis, M. Ailion, E. M. Jorgensen, Improved *Mos1*-mediated transgenesis in *C. elegans*. *Nat. Methods* **9**, 117–118 (2012).

71. A. C. Hart, "Behavior" in *WormBook*, The C. elegans Research Community, Ed. (WormBook, 2006).

72. J. Schindelin, I. Arganda-Carreras, E. Frise, V. Kaynig, M. Longair, T. Pietzsch, S. Preibisch, C. Rueden, S. Saalfeld, B. Schmid, J. Y. Tinevez, D. J. White, V. Hartenstein, K. Eliceiri, P. Tomancak, A. Cardona, Fiji: An open-source platform for biological-image analysis. *Nat. Methods* **9**, 676–682 (2012).

73. C. F. Hryc, M. L. Baker, Beyond the backbone: The next generation of pathwalking utilities for model building in cryoEM density maps. *Biomolecules* **12**, 773 (2022).

74. G. Fan, M. R. Baker, L. E. Terry, V. Arige, M. Chen, A. B. Seryshev, M. L. Baker, S. J. Ludtke, D. I. Yule, I. I. Serysheva, Conformational motions and ligand-binding underlying gating and regulation in IP_3R channel. *Nat. Commun.* **13**, 6942 (2022).

75. G. M. Torrie, J. P. Valleau, Nonphysical sampling distributions in Monte Carlo free-energy estimation: Umbrella sampling. *J. Comput. Phys.* **23**, 187–199 (1977).

76. S. Kumar, D. Bouzida, R. H. Swendsen, P. A. Kollman, J. M. Rosenberg, The weighted histogram analysis method for free-energy calculations on biomolecules. 1. The method. *J. Comput. Chem.* **13**, 1011–1021 (1992).

77. B. Isralewitz, M. Gao, K. Schulten, Steered molecular dynamics and mechanical functions of proteins. *Curr. Opin. Struct. Biol.* **11**, 224–230 (2001).

78. J. Abramson, J. Adler, J. Dunger, R. Evans, T. Green, A. Pritzel, O. Ronneberger, L. Willmore, A. J. Ballard, J. Bambrick, S. W. Bodenstein, D. A. Evans, C. C. Hung, M. O'Neill, D. Reiman, K. Tunyasuvunakool, Z. Wu, A. Zemgulyte, E. Arvaniti, C. Beattie, O. Bertolli, A. Bridgland, A. Cherepanov, M. Congreve, A. I. Cowen-Rivers, A. Cowie, M. Figurnov, F. B. Fuchs, H. Gladman, R. Jain, Y. A. Khan, C. M. R. Low, K. Perlin, A. Potapenko, P. Savy, S. Singh, A. Stecula, A. Thillaisundaram, C. Tong, S. Yakneen, E. D. Zhong, M. Zielinski, A. Zídek, V. Bapst, P. Kohli, M. Jaderberg, D. Hassabis, J. M. Jumper, Accurate structure prediction of biomolecular interactions with AlphaFold 3. *Nature* **630**, 493–500 (2024).

79. S. Jo, J. B. Lim, J. B. Klauda, W. Im, CHARMM-GUI Membrane Builder for mixed bilayers and its application to yeast membranes. *Biophys. J.* **97**, 50–58 (2009).

80. J. S. Hub, B. L. de Groot, D. van der Spoel, g_wham-A free weighted histogram analysis implementation including robust error and autocorrelation estimates. *J. Chem. Theory Comput.* **6**, 3713–3720 (2010).

81. M. J. Abraham, T. Murtola, R. Schulz, S. Páll, J. C. Smith, B. Hess, E. Lindahl, GROMACS: High performance molecular simulations through multi-level parallelism from laptops to supercomputers. *SoftwareX* **1-2**, 19–25 (2015).

82. H. J. C. Berendsen, J. P. M. Postma, W. F. Vangunsteren, A. Dinola, J. R. Haak, Molecular-dynamics with coupling to an external bath. *J. Chem. Phys.* **81**, 3684–3690 (1984).

83. M. Parrinello, A. Rahman, Polymorphic transitions in single-crystals—A new molecular-dynamics method. *J. Appl. Phys.* **52**, 7182–7190 (1981).

84. G. Bussi, D. Donadio, M. Parrinello, Canonical sampling through velocity rescaling. *J. Chem. Phys.* **126**, 014101 (2007).

85. B. Hess, P-LINCS: A parallel linear constraint solver for molecular simulation. *J. Chem. Theory Comput.* **4**, 116–122 (2008).

86. T. Darden, D. York, L. Pedersen, Particle mesh Ewald—An N.Log(N) method for Ewald sums in large systems. *J. Chem. Phys.* **98**, 10089–10092 (1993).

87. H. J. C. Berendsen, J. R. Grigera, T. P. Straatsma, The missing term in effective pair potentials. *J. Phys. Chem.* **91**, 6269–6271 (1987).

88. K. Lindorff-Larsen, S. Piana, K. Palmo, P. Maragakis, J. L. Klepeis, R. O. Dror, D. E. Shaw, Improved side-chain torsion potentials for the Amber ff99SB protein force field. *Proteins* **78**, 1950–1958 (2010).

89. C. J. Dickson, R. C. Walker, I. R. Gould, Lipid21: Complex lipid membrane simulations with AMBER. *J. Chem. Theory Comput.* **18**, 1726–1736 (2022).

90. M. Macchiagodena, M. Pagliai, C. Andreini, A. Rosato, P. Procacci, Upgrading and validation of the AMBER force field for histidine and cysteine zinc(II)-binding residues in sites with four protein ligands. *J. Chem. Inf. Model.* **59**, 3803–3816 (2019).

91. I. S. Joung, T. E. Cheatham, Determination of alkali and halide monovalent ion parameters for use in explicitly solvated biomolecular simulations. *J. Phys. Chem. B* **112**, 9020–9041 (2008).

92. J. A. Delgado, V. Wineman-Fisher, S. Pandit, S. Varma, Inclusion of high-field target data in AMOEBA's calibration improves predictions of protein-ion interactions. *J. Chem. Inf. Model.* **62**, 4713–4726 (2022).

93. J. M. Delgado, P. R. Nagy, S. Varma, Polarizable AMOEBA model for simulating Mg^{2+}·protein·nucleotide complexes. *J. Chem. Inf. Model.* **64**, 378–392 (2024).
